# Integration of single-cell and bulk RNA sequencing data identified an aortic dissection-enriched ANGPTL4+ macrophage subpopulation

**DOI:** 10.3389/fimmu.2026.1874233

**Published:** 2026-08-28

**Authors:** Zhenghao Li, Haonan Chen, Xuanyu Liu, Yue Shao, Yinding Peng, Qingchen Wu, Haoming Shi, Cheng Zhang

**Affiliations:** 1 Department of Cardiothoracic Surgery, The First Affiliated Hospital of Chongqing Medical University, Chongqing, China; 2 Department of Urology, The First Affiliated Hospital of Chongqing Medical University, Chongqing, China

**Keywords:** ANGPTL4, aortic dissection, bulk RNA sequencing, macrophage, single-cell RNA sequencing

## Abstract

**Background:**

Macrophages play an indispensable role in the pathogenesis of aortic dissection (AD). However, the underlying molecular and cellular mechanisms remain incompletely understood.

**Methods:**

We integrated six publicly available single-cell RNA sequencing (scRNA-seq) datasets and constructed an atlas of AD, identifying an AD-enriched ANGPTL4+ macrophage subpopulation. Subsequent comprehensive bioinformatic analyses explored its potential mechanisms in AD. Using machine learning (ML) algorithms on integrated bulk RNA sequencing (bulk RNA-seq) data, we screened the signature genes of ANGPTL4+ macrophage and constructed a clinical prediction model. The correlation between the proportion of ANGPTL4+ macrophage and clinical features was investigated after CIBERSORTx deconvolution. Finally, we performed *in vitro* experiments to validate our findings.

**Results:**

Bioinformatic analyses revealed that ANGPTL4+ macrophage was predominantly present in AD, exhibited a distinct glycolytic phenotype, represented an early monocyte-derived macrophage state in the dissected aorta, predicted to be regulated by PPARG, and showed inferred ligand-receptor interactions with structural cells through ANGPTL and SPP1 signaling pathways. ML algorithms identified ANGPTL4, PHLDA1, and SERPINE1 as signatures of ANGPTL4+ macrophage. The proportion of ANGPTL4+ macrophage was positively associated with clinical metrics like monocyte count. *In vitro* experiments validated the remarkable enrichment of ANGPTL4+ macrophage and considerable up-regulation and co-localization of the signatures.

**Conclusions:**

In-depth investigation into the potential mechanisms of ANGPTL4+ macrophage, an AD-enriched macrophage subpopulation, and its signatures ANGPTL4, PHLDA1, and SERPINE1, provides novel insights into the pathogenesis and prospective therapeutic targets of AD.

## 1 Introduction

Aortic dissection (AD), particularly Stanford type A AD (TAAD), is a critical cardiovascular emergency with extremely high mortality characterized by a tear of the aorta, leading to separation of the tunica media and tunica intima (1, 2). Despite advances in surgical treatment, which is the most effective therapeutic option for AD, alternative pharmacological treatments are lacking (3). Previous studies have implicated aortic wall degeneration and inflammation, including loss of smooth muscle cells (SMC), extracellular matrix (ECM) degradation, and immune cell infiltration, which occurs in the pathogenesis of AD; however, the underlying molecular and cellular mechanisms remain incompletely understood (4, 5). Elucidating systemic cellular pathogenic mechanisms of AD may provide novel strategies for early prediction and targeted therapy.

Macrophages in aortic tissue are predominantly derived from circulating monocytes, with some subsets representing tissue-resident macrophages (TRM). Different macrophage subsets mediate complex processes of deterioration or renovation through ECM remodeling, inflammatory regulation, and tissue repair (5, 6). Macrophages exemplify a highly heterogeneous population, with complexity manifested by distinct cytokine secretion patterns, differential surface marker expression, and divergent transcriptional profiles. Single-cell RNA sequencing (scRNA-seq) can greatly aid our comprehension of cellular heterogeneity in aorta, enabling the identification and interpretation of distinct cell subpopulations under different disease states and providing potential therapeutic targets. For instance, IL1B+ inflammatory macrophage, which differentiates from classical monocytes, is significantly enriched in AD (7). And BASP1+ monocyte subset infiltrates aortic tissue early during the pathogenesis of AD, inducing apoptosis of SMCs and endothelial cells (EC) (8). Nevertheless, an integration of publicly available scRNA-seq datasets combined with bulk transcriptomic profiles and experimental validation to identify AD-enriched macrophage subsets and their clinical relevance has not been systematically performed. Herein, we aim to extensively explore AD from a single-cell perspective, with a particular focus on the heterogeneity and functional diversity of macrophages.

In this study, we combined six scRNA-seq datasets of TAAD with bulk RNA-seq datasets from both public databases and in-house transcriptomes to create a detailed single-cell atlas of dissected human aorta. We discovered an AD-enriched macrophage subpopulation marked by differential high expression of ANGPTL4 with a glycolytic phenotype for the first time. This subset likely represented an early monocyte-derived macrophage state, predicted to be regulated by transcription factor (TF) PPARG, and showed inferred communications with other cell types through ANGPTL and SPP1 signaling pathways. Machine learning (ML) identified ANGPTL4, PHLDA1, and SERPINE1 as key markers of this subset. We then exploratively correlated the deconvoluted proportion of macrophage subsets in the immune microenvironment of the dissected aorta with clinical parameters, founding that the proportion of ANGPTL4+ macrophage was closely associated with metrics like monocyte count. Experimental validation confirmed the increased presence of ANGPTL4+ macrophage and elevated expression of identified signatures in AD. Our findings offer a single-cell resource for AD and highlight ANGPTL4+ macrophage as a potential therapeutic target.

## 2 Methods

### 2.1 Data acquisition

We searched “aortic dissection” in the Gene Expression Omnibus (GEO) database (https://www.ncbi.nlm.nih.gov/geo/) (9), restricting top organisms to “Homo sapiens” and entry type to “Series”, which yielded six scRNA-seq datasets and six bulk RNA-seq datasets (**Table 1**). For scRNA-seq datasets, the raw count data were downloaded. For bulk RNA-seq datasets, if the study type was expression profiling by high-throughput sequencing, the raw count matrices were downloaded, otherwise the Transcripts Per Million (TPM) matrices were downloaded.

**Table 1:** The publicly available transcriptome sequencing datasets included in this study.

Data type	Dataset	Accession ID	Number of normal samples	Number of AD samples
scRNA-seq	Li et al. (10)	GSE155468	3	0
	Zhang et al. (11)	GSE213740	3	6
	Luo et al. (12)	GSE222318	5	9
	Inoue et al. (7)	GSE224559	0	1
	Cai et al. (13)	GSE226492	3	3
	Zhao et al. (14)	GSE254132	3	3
bulk RNA-seq	Pan et al. (15)	GSE52093	5	7
	Kimura et al. (16)	GSE98770	5	6
	Zhou et al. (17)	GSE147026	4	4
	Zhou et al. (18)	GSE153434	10	10
	Huang et al. (19)	GSE190635	4	4
	Wang et al. (20)	GSE241265	3	3

AD, aortic dissection; scRNA-seq, single-cell RNA sequencing; bulk RNA-seq, bulk RNA sequencing.

### 2.2 Single-cell RNA sequencing analyses

#### 2.2.1 Quality control and data processing

We collected six publicly available scRNA-seq datasets of TAAD, comprising 17 normal and 22 dissected aortic samples. Raw sequencing data were loaded into R environment using the Seurat package (21), and the proportions of mitochondrial genes and erythrocyte genes were calculated. For each sample, doublets were identified using the DoubletFinder package, employing default parameters and a doublet rate estimated based on input cell number after pre-processing (22). To remove ambient RNA contamination, we used the DecontX package to add the corrected raw count matrices into the Seurat object (23). We applied cell filtering criteria to retain cells with: 1) number of genes > 200 and < the 95th percentile of maximum genes across all cells; 2) number of counts < the 95th percentile of maximum counts across all cells; 3) mitochondrial gene proportion < 10%; 4) erythrocyte gene proportion < 5%; 5) singlet (non-doublet); and 6) contamination < 0.2. We then processed the raw count data using a standard workflow, including normalization, identification of highly variable features, linear transformation (scaling), and principal component analysis (PCA) dimensionality reduction. According to Seurat V5, the IntegrateLayers function can be used to perform data integration across different batches. We set “method = HarmonyIntegration” to employ the Harmony algorithm for data integration and batch effect correction, naming the integrated dimension “harmony” (24). The first 10 principal components, the harmony reduction, and a resolution of 0.6 were used for primary clustering, which involved shared nearest-neighbor (SNN) graph construction and Louvain algorithm clustering. Finally, we performed uniform manifold approximation and projection (UMAP) dimensionality reduction using the first 10 principal components for visualization.

#### 2.2.2 Cell annotation

##### 2.2.2.1 Level 1 annotation

We performed cell annotation based on canonical aortic cell markers from previous scRNA-seq atlases of artery (10, 12, 25), including SMCs (MYH11, ACTA2, MYL9), fibroblasts (DCN, COL1A1, COL1A2), ECs (PECAM1, VWF, PLVAP), Schwann cells (MPZ, S100B, SOX10), myeloid cells (LYZ, CD14, CD68), neutrophils (CSF3R, FCGR3B, S100A12), mast cells (CPA3, KIT, TPSB2), T cells (CD3D, CD3E, CD2), natural killer (NK) cells (KLRD1, NKG7, XCL1), B cells (CD79A, CD79B, MS4A1), plasma cells (MZB1, JCHAIN, IGKC), and proliferation cells (MKI67, TOP2A, CDC20). The FindAllMarkers function was used to calculate differentially expressed genes (DEGs) for each cell cluster, with parameters set to logfc.threshold = 0.25, min.pct = 0.25, and only.pos = TRUE.

##### 2.2.2.2 Level 2 annotation

We performed secondary dimensionality reduction and clustering on structural cells (SMCs, fibroblasts, and ECs), myeloid cells (monocytes and macrophages), and lymphocytes (T cells and B cells). The FindAllMarkers function was used to calculate DEGs for each cell cluster. The AddModuleScore function was employed to evaluate the expression level of a given gene set in individual cells, thereby deriving corresponding cell scores. To elucidate the biological functions of each cell cluster, we obtained gene ontology (GO) and Kyoto Encyclopedia of Genes and Genomes (KEGG) gene sets from the Molecular Signatures Database (MSigDB) (https://www.gsea-msigdb.org/gsea/msigdb) (26), and then performed gene set enrichment analysis (GSEA) using the clusterProfiler package (27). According to the specifically expressed genes and the executed biological functions of each cell cluster, we conducted more elaborate cell annotation.

#### 2.2.3 Quantification of enrichment and specificity of cell types

To quantify the enrichment of cell clusters across different groups, we compared the observed and expected cell numbers in each cluster by calculating *R*_o/e_ value, and *R*_o/e_ > 1 indicated that one cell cluster was enriched in a specific group (28). We also calculated entropy value of each cluster to clarify whether one cell cluster was classified as specific, and entropy value < 0.625 indicated a specific cell cluster (29).

#### 2.2.4 Pseudo-time trajectory deduction

Pseudo-time analysis is a method used to measure the progress of individual cells through processes. We performed pseudo-time analysis on the unlabeled scRNA-seq data using the monocle2 package (30). This analysis constructs the temporal trajectory of cell development and infers the dynamic evolutionary processes of cell fate by selecting highly variable genes, performing dimensionality reduction, constructing a trajectory tree, and ordering cells along the pseudo-time. We also conducted slingshot analysis to infer cell lineages and pseudo-times (31).

#### 2.2.5 Metabolism activity quantification

We performed Metabolism activity analysis using the scMetabolism package (32). This package incorporates metabolic pathway gene sets from the KEGG and Reactome databases and employs algorithms such as VISION to quantify and visualize the metabolism activity levels of cells, facilitating comparison of metabolic states across different cell subpopulations (33). And we further validated glycolysis phenotype using AUCell, UCell, ssgsea, GSVA, AddModuleScore and zscore methods.

#### 2.2.6 Gene regulatory network identification

Single-cell regulatory network inference and clustering (SCENIC) is an algorithm used to infer gene regulatory networks (GRNs) from scRNA-seq data (34). It incorporates TF motif analysis to validate statistically inferred co-expression networks, thereby identifying highly reliable regulons. The workflow involves five main steps. First, soft gene filter is applied to exclude genes that are expressed at an extremely low level or in only a minority of cells. Second, potential TF targets are inferred from the expression data using GENIE3 or GRNBoost. Third, potential direct-binding targets are selected based on DNA-motif analysis with RcisTarget. Fourth, regulon activity is scored in individual cells using AUCell. Last, stable cell states are identified based on the activity of their GRNs.

#### 2.2.7 Cell-cell communication characterization

CellChatDB is a ligand-receptor interaction database contains approximately 3,300 validated molecular interactions, including secrete autocrine/paracrine signaling interactions, extracellular matrix (ECM)-receptor interactions, cell-cell contact interactions, and non-protein signaling. By using an integrative approach that combines manifold analyses, CellChat analysis can quantitatively characterize inferred cell-cell communication networks from given scRNA-seq data based on CellChatDB (35). Additionally, CellChat enables comparative analysis of cell-cell communication across different conditions and identifies signaling pathways and cell populations. We used the CellChat package to analyze and uncover discrepancies in cell-cell communication between normal and AD.

### 2.3 Bulk RNA sequencing analysis

#### 2.3.1 Human aortic specimen collection

We collected two batches of dissected and normal aortic specimens and submitted to BGI Genomics Co., Ltd. for bulk RNA-seq. Dissected aortic specimens were obtained from patients with TAAD who were admitted to the Department of Cardiothoracic Surgery at the First Affiliated Hospital of Chongqing Medical University between 2023 and 2025 and underwent surgical treatment. Normal aortic specimens were obtained from healthy donors during the same period. Donors who were younger than 18 years, had a history of aortic or major vascular diseases, or had severe infections were excluded. This study was conducted in accordance with the principles of the Declaration of Helsinki and was approved by the Ethics Committee of the First Affiliated Hospital of Chongqing Medical University. All participants provided written informed consent.

#### 2.3.2 Data processing and integration

We totally collected 40 normal and 59 dissected aortic samples from public and our cohorts. For high throughput sequencing data, after removing genes that were not expressed in more than half of the samples, variance stabilizing transformation and normalization were performed on raw count data using the DESeq2 package (36). Others, normalization was conducted after removing genes and log 2 transformation. After integrating the gene expression matrices, batch effects were adjusted using the sva package (37).

#### 2.3.3 Differential expression analysis

Differential expression analysis was performed using the limma package (38), which is based on a linear model and employs an empirical Bayes method to moderate the estimates of gene expression differences. The Benjamini-Hochberg method was applied to adjust the P-values for multiple testing to control the false discovery rate. Genes with an absolute log 2 fold change greater than 1 and an adjusted P-value less than 0.05 were considered as DEGs.

#### 2.3.4 Machine learning

We performed ML on the integrated gene expression matrix using the caret package to construct a predictive model for accurately distinguishing between AD patients and normal individuals (39). Following the caret tutorial, after identifying and eliminating near zero variables (variables with one unique value), highly correlated variables (r > 0.9), and linearly dependent variables, we pre-processed the gene expression matrix, including centering and scaling. The samples were then randomly divided into training and test sets at a 70% and 30% ratio. A repeated 5×10-fold cross-validation resampling method was used for parameter tuning, with receiver operating characteristic (ROC) as the metric. A total of 102 ML algorithms for classification were utilized and the optimal algorithms with the best performance was selected.

#### 2.3.5 Model interpretability analysis

We performed dataset-level explanation using the kernelshap package. The permutation Shapley additive explanations (SHAP) algorithm was employed to explain the ML models by quantifying the contribution of individual variables to prediction (40). Significant genes were ranked by their average SHAP values. The top 20 genes from different predictive models were intersected to identify genes that contributed significantly across models. The shapviz package was used for SHAP visualization (41).

#### 2.3.6 Clinical prediction model construction

We overlapped genes with the absolute SHAP values of the best-performing ML algorithms and DEGs of AD to obtain the final signatures. ROC curves of each individual signature were depicted and area under the curve (AUC) was calculated to evaluate the predictive accuracy. A logistic regression model was then constructed based on all signatures for clinical prediction, and calibration curves and clinical decision curves were used to assess the consistency of model fitting and clinical utility.

#### 2.3.7 Cell proportion estimation

We generated a gene signature matrix of macrophages from the integrated scRNA-seq of AD. Subsequently, we performed deconvolution on the bulk RNA-seq data from our cohort using CIBERSORTx tool (https://cibersortx.stanford.edu/) with the default parameters and 100 permutations to infer the proportions of different macrophage subpopulations in each sample, enabling further clinical analysis (42).

#### 2.3.8 Single sample gene set enrichment analysis

We collected glycolysis-related genes from the MsigDB database. Based on the expression levels of these genes, we calculated a glycolysis score for each sample using the single sample gene set enrichment analysis (ssGSEA) algorithm to characterize the activation level of the glycolysis pathway.

#### 2.3.9 Exploratory clinical correlation analysis

We collected the clinical information of the 25 AD patients in our sequencing cohort, including baseline characteristics, preoperative laboratory tests, and length of hospital stay. Subsequently, we calculated the Spearman correlations among gene signatures, macrophage proportions, glycolysis scores, and clinical information to preliminarily explore the potential clinical significance of macrophage proportions.

### 2.4 Experiments

#### 2.4.1 Immunofluorescence

Immunofluorescence was performed on paraffin-embedded aortic tissue sections. After deparaffinization, antigen retrieval, and blocking, sections were incubated with primary antibodies against CD68 (Abcam 53444), ANGPTL4 (Proteintech 67577), PHLDA1 (Abcam 133654), and SERPINE1 (Abcam 222754), followed by fluorochrome-conjugated secondary antibodies. Nuclei were counterstained with DAPI, and ANGPTL4+ macrophage was quantified.

#### 2.4.2 Quantitative real-time polymerase chain reaction

Total RNA was extracted from aortic tissues using TRIzol reagent, and reverse transcription was performed. Quantitative real-time polymerase chain reaction (qRT-PCR) was conducted using SYBR Green Master Mix with specific primers for ANGPTL4, PHLDA1, and SERPINE1, with ACTB as an internal control (**Supplementary Table 1**). RNA samples with an OD260/OD280 ratio ranging from 1.8 to 2.0 were considered to have acceptable purity. Relative mRNA expression was then calculated using 2^−ΔΔCt^ method.

#### 2.4.3 Western blot

For western blot (WB), aortic tissue lysates were separated by SDS-PAGE, transferred onto PVDF membranes, and incubated with primary antibodies against ANGPTL4, PHLDA1, SERPINE1, and GAPDH (Abclonal), followed by HRP-conjugated secondary antibodies. Protein bands were visualized using enhanced chemiluminescence and quantified by densitometry using ImageJ software. The relative protein expression level was calculated as the ratio of the grayscale intensity of the target protein band to that of the internal reference band.

### 2.5 Statistical analysis

R (version 4.3.3) and GraphPad Prism (version 8) were used for statistical analyses and visualization of bioinformatics and experiments, respectively. Continuous variables were presented as means with standard deviations or medians with interquartile ranges. Differences between two groups were assessed using the Wilcoxon rank-sum test (non-normally distributed data) or the t-test (normally distributed data).

## 3 Results

### 3.1 Integrated single-cell landscape of TAAD

#### 3.1.1 Integration of publicly available scRNA-seq datasets

**Figure 1:**
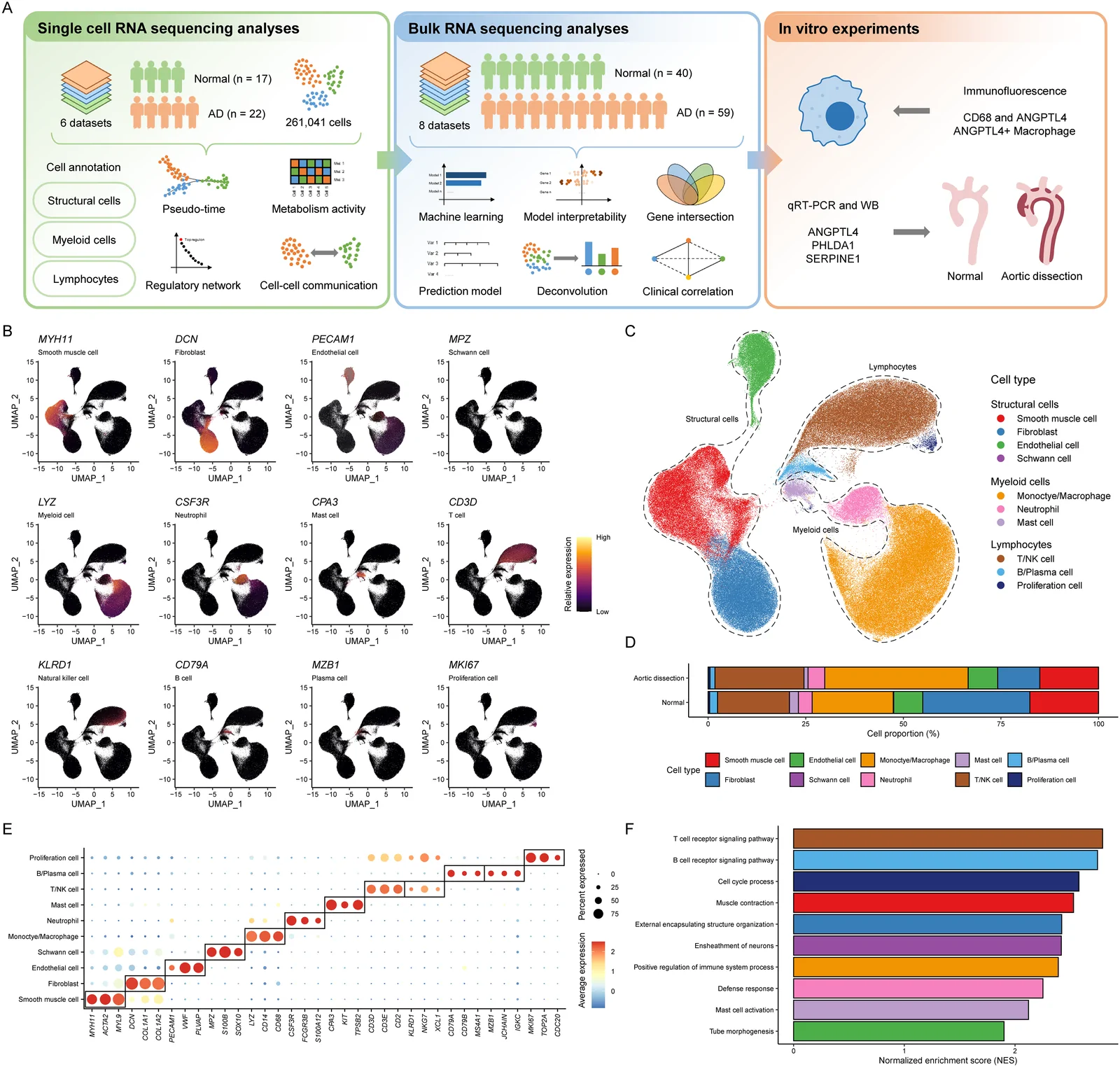
Study workflow and level 1 annotation on integrated single-cell landscape of TAAD. **(A)** The workflow of this study. **(B)** Distribution of aortic cell markers on UMAP plots. **(C)** UMAP plot of level 1 cell annotation for integrated single-cell landscape of TAAD. **(D)** Bar plot of cell type proportions in normal and AD samples. **(E)** Expression profiles of markers for each cell type. **(F)** Top enriched cellular BP for each cell type. TAAD, Stanford type A aortic dissection; UMAP, uniform manifold approximation and projection; BP, biological process.

The workflow of this study is shown in **Figure 1A**. We collected and integrated the six publicly available scRNA-seq datasets of TAAD. After quality control, a total of 261,041 cells from 17 normal and 22 dissected aortic samples were included (**Supplementary Figure 1A**). Subsequently, a standard data pre-processing workflow was performed, we use Harmony algorithm to correct batch effect and UMAP method to visualize, ultimately yielding 21 cell clusters (**Supplementary Figure 1B**). Based on canonical cell markers for aortic tissue described in the literature (**Figure 1B**) (10, 12), we identified three main categories comprising 10 distinct cell types: structural cells, including SMCs, fibroblasts, ECs, and Schwann cells; myeloid cells, including monocytes/macrophages, neutrophils, and mast cells; and lymphocytes, including T/NK cells, B/Plasma cells, and proliferation cells (**Figure 1C**). Among these, SMCs and fibroblasts were decreased in AD, whereas monocytes/macrophages were increased (**Figure 1D**), suggesting the loss of structural cells and infiltration of immune cells in AD. The gene expression profiles and enriched biological processes of different cell types revealed the cellular diversity in AD (**Figures 1E, F**), which is consistent with current consensus.

#### 3.1.2 Identification of structural cells

**Figure 2:**
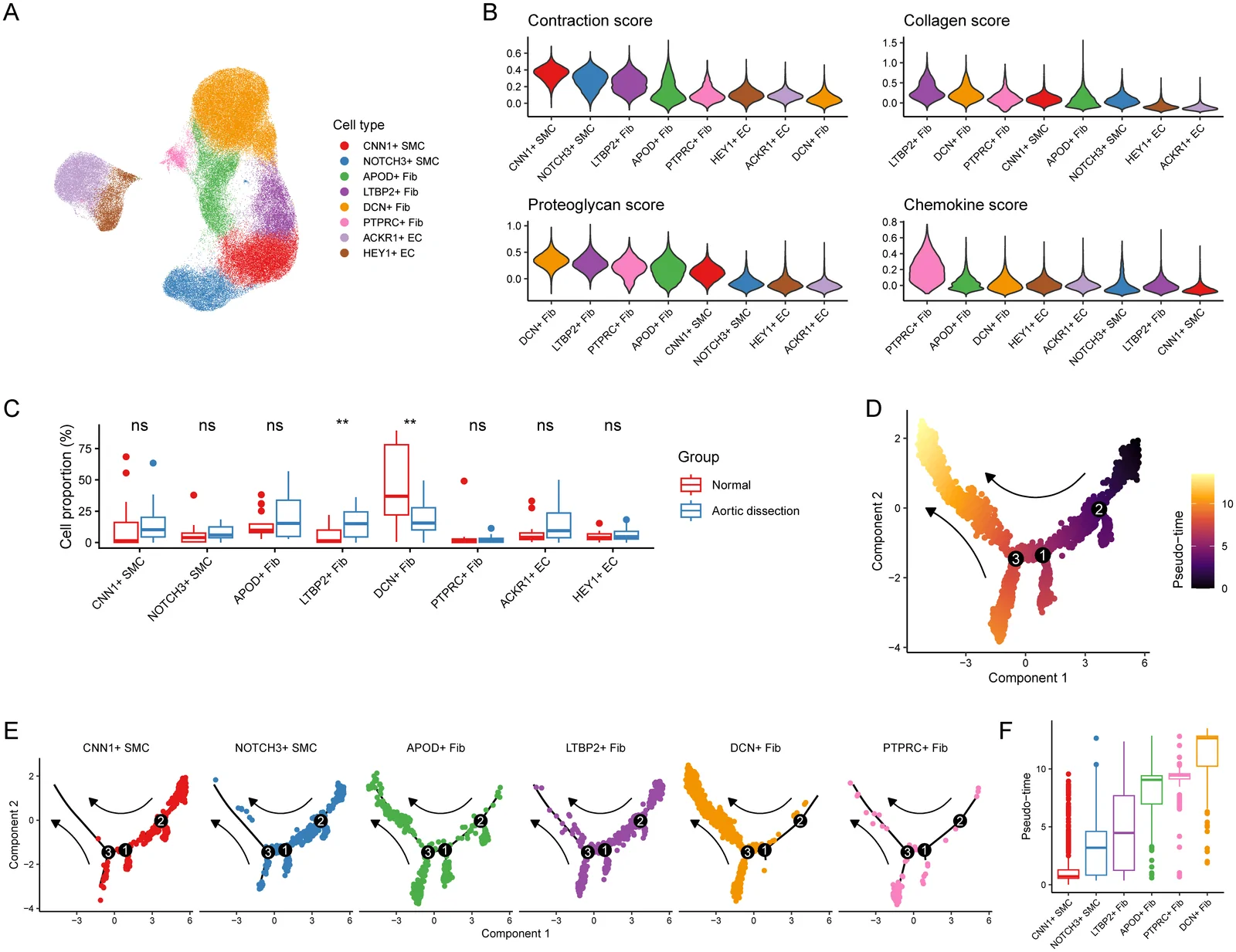
Level 2 cell annotation and pseudo-time trajectory analysis of structural cells. **(A)** UMAP plot of level 2 cell annotated structural cells after secondary dimensionality reduction and clustering. **(B)** Gene module scores for each subpopulation, ranked in descending order by average. **(C)** Differences in proportions of each subpopulation between normal and AD samples (** P < 0.01). **(D)** Pseudo-time analysis classified SMCs, fibromyocytes, and fibroblasts into seven states, with the developmental trajectory from right to left. **(E)** Distribution of each subpopulation along the pseudo-time trajectory. **(F)** Pseudo-time of each subpopulation, ranked in ascending order by average. UMAP, uniform manifold approximation and projection; AD, aortic dissection; SMC, smooth muscle cell.

We extracted primary SMCs, fibroblasts, and ECs for secondary dimensionality reduction and clustering (dims = 1:10, res = 0.3), which yielded 10 cell clusters. These were ultimately identified as four categories comprising 8 cell types, including two clusters of SMCs, 2 clusters of fibromyocytes, 2 clusters of fibroblasts, and 2 clusters of ECs (**Figure 2A**; **Supplementary Figure 1C**). We performed level 2 cell annotation based on their expression profiles of up-regulated DEGs and enriched biological processes (BPs) and pathways (**Supplementary Figures 1D–F**). To better compare discrepancies in cellular functions, we calculated contraction score, ECM score (including collagen and proteoglycan score), and chemokine score for each cell subpopulation (**Figure 2B**). The proportion of LTBP2+ Fib was significantly elevated in AD, while that of DCN+ Fib was significantly reduced (**Figure 2C**).

##### 3.1.2.1 Smooth muscle cells (Contractile SMCs)

CNN1+ SMC exhibited high expression of contraction-related genes such as MYH11, ACTG2, and CNN1, and enriched in BPs and pathways mainly including smooth muscle contraction and muscle system processes, with the highest contraction score and a moderate ECM score. The second cluster, designated NOTCH3+ SMC, showed high expression of NOTCH3 and other contraction-regulating genes, with BPs and pathways primarily enriched in muscle contraction and regulation of muscle contraction, and also exhibiting a high contraction score. Previous studies have shown that NOTCH3 signaling can be activated to maintain the contractile phenotype of vascular SMCs (43, 44).

##### 3.1.2.2 Fibromyocytes (Synthetic SMCs)

Both two fibromyocyte clusters co-expressed contraction-related and ECM-related genes, exhibiting moderate contraction and ECM scores. They were respectively named according to their most remarkably up-regulated DEGs (APOD+ Fib and LTBP2+ Fib). APOD+ Fib principally enriched in BPs and pathways related to cytoplasmic translation, whereas LTBP2+ Fib was associated with ECM receptor interaction and cell adhesion. Targeted inhibition of LTBP2 can attenuate vascular and myocardial remodeling and enhance the normal phenotypic expression in vascular SMCs and cardiomyocytes (45, 46), suggesting that LTBP2+ Fib may represent an aberrant outcome of SMC phenotypic switching in AD, which may serve as a potential therapeutic target for AD.

##### 3.1.2.3 Fibroblasts

DCN+ Fib represent the classical fibroblast subset, characterized by ECM-related genes such as DCN, LUM, and COL1A1, and enrichment in collagen metabolic process and regulation of ECM organization, as well as the lowest contraction score and the highest ECM score. PTPRC+ Fib additionally exhibited high expression of inflammation-related genes such as IL1B, CCL5, and PTPRC.

##### 3.1.2.4 Endothelial cells

We performed level 2 annotation on ECs according to existing endothelial cell single-cell atlases (47, 48). Venous EC (ACKR1+ EC) was identified by ACKR1, SELE, and VCAM1, while arterial EC (HEY1+ EC) was identified by HEY1, SEMA3G, and EFNB2. The cellular functions of the two subsets also differed. ACKR1+ EC mainly enriched in peptide antigen assembly, whereas HEY1+ EC participated more in endothelium development and establishment of endothelial barrier.

##### 3.1.2.5 Deciphering SMC phenotypic switching at single-cell resolution

We performed pseudo-time trajectory inference on SMCs, fibromyocytes, and fibroblasts. All cells were assigned to seven states, with SMCs primarily located at the beginning of the trajectory, fibroblasts at the end, and fibromyocytes positioned in between, suggesting a developmental trajectory of “SMC-fibromyocyte-fibroblast” (**Figures 2D–F**). These results effectively recapitulate the pathophysiological process of phenotypic switching of SMCs from contractile to synthetic phenotype in AD at single-cell resolution. The observation that the expression levels of contraction-related genes and score gradually decreased along the pseudo-time, whereas ECM-related genes and score progressively increased, further reinforce our results (**Supplementary Figure 1G**).

#### 3.1.3 Identification of myeloid cells

**Figure 3:**
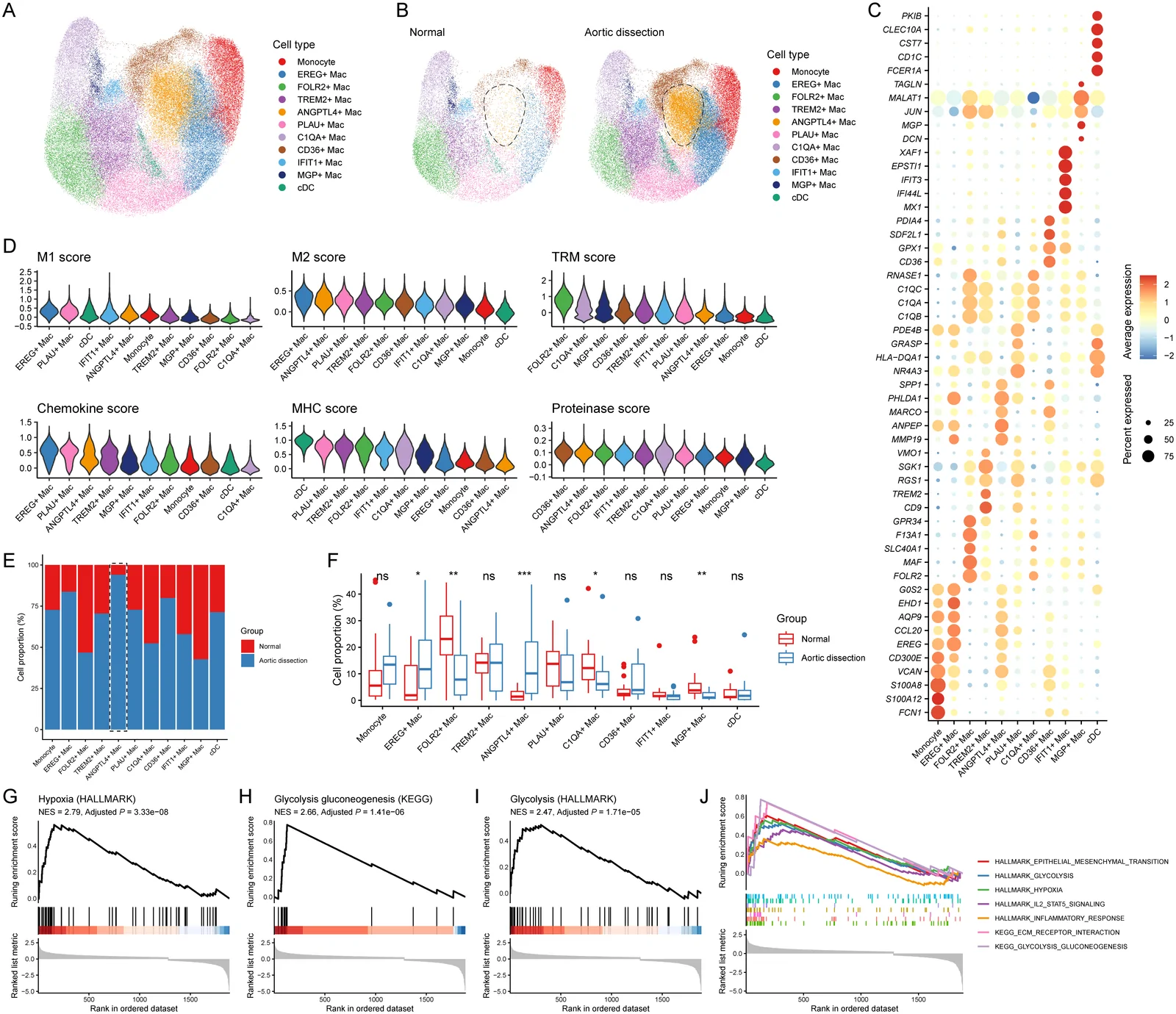
Level 2 cell annotation of myeloid cells. **(A)** UMAP plot of level 2 cell annotated myeloid cells after secondary dimensionality reduction and clustering. **(B)** UMAP plots of level 2 cell annotations of myeloid cells in normal and AD samples. **(C)** Top 5 up-regulated DEGs for each subpopulation. **(D)** Gene module scores for each subpopulation, ranked in descending order by average. **(E)** Proportions of each subpopulation originated from normal or AD samples. **(F)** Differences in proportions of each subpopulation between normal and AD samples (*P < 0.05, **P < 0.01, ***P < 0.001). **(G)** Enrichment of ANGPTL4+ Mac in the HALLMARK hypoxia pathway. **(H)** Enrichment of ANGPTL4+ Mac in the KEGG glycolysis/gluconeogenesis pathway. **(I)** Enrichment of ANGPTL4+ Mac in the HALLMARK glycolysis pathway. **(J)** The significantly enriched HALLMARK and KEGG pathways of ANGPTL4+ Mac. UMAP, uniform manifold approximation and projection; AD, aortic dissection; DEG, differentially expressed gene; KEGG, Kyoto Encyclopedia of Genes and Genomes.

We extracted level 1 monocytes/macrophages for secondary dimensionality reduction and clustering (dims = 1:10, res = 0.6), which yielded 12 cell clusters. These were ultimately identified as three categories comprising 11 cell types, including 1 cluster of monocytes, 9 clusters of macrophages, and 1 cluster of dendritic cells (DC) (**Figures 3A, B**). Consistent with our previous strategies, up-regulated DEGs, GSEA results, and gene module scores were used for level 2 annotation (**Figures 3C, D**; **Supplementary Figures 2A, B**). We selected cell state gene modules including M1, M2, and TRM, along with cell function gene modules including chemokines, major histocompatibility complex (MHC), and proteinase. The proportions of EREG+ Mac, FOLR2+ Mac, ANGPTL4+ Mac, C1QA+ Mac, and MGP+ Mac significantly differed between normal and AD samples (**Figures 3E, F**). Notably, ANGPTL4+ Mac was almost exclusively originated from AD (~94%), suggesting that it may represent an AD-enriched macrophage subset.

##### 3.1.3.1 Macrophages

EREG+ Mac highly expressed EREG and CCL20, exhibited the highest M1, M2, and chemokine scores, suggesting that it may function as an intermediate transitional state in macrophage polarization or act as local response amplifiers, recruiting more immune cell infiltration. The enriched BPs and pathways mainly included cytokine-cytokine receptor interaction and the cytokine mediated signaling pathway. FOLR2+ Mac was characterized by TRM-related genes such as FOLR2, C1QA, and LYVE1, displayed the highest TRM score, and enriched in complement and coagulation cascades and antigen processing and presentation, indicating that it may represent TRM in AD. TREM2+ Mac highly expressed TREM2 and CD9 with moderate M1 and M2 scores, and were functionally associated with antigen processing and presentation and peptide antigen assembly. This cell subset prominently appeared in atherosclerotic aorta of both human and mice, where it was considered as foamy macrophage (49, 50). And TREM2 served as a promising potential biomarker for atherosclerosis progression (51). The fourth macrophage cluster was named ANGPTL4+ Mac, which may be a potential AD-enriched macrophage subset, with high expression of ANGPTL4, SPP1, and MMP19, and relatively high M2 and proteinase scores. After reviewing the top 20 differentially up-regulated genes, we selected ANGPTL4 to name this subset, which didn’t express in majority of the other macrophages (**Supplementary Figure 2C**). Noteworthily, ANGPTL4+ Mac appeared to engage in multiple metabolic processes, including glycolysis/gluconeogenesis and nucleoside diphosphate metabolic process. To better understand the biological functions of this subset, we performed GSEA using the HALLMARK and KEGG pathway gene sets. The results revealed significant enrichment of pathways such as hypoxia and glycolysis/gluconeogenesis (**Figures 3G–J**), suggesting that in local immune microenvironment of AD, this M2-like macrophage underwent metabolic reprogramming driven by hypoxia, resulting in a pronounced glycolytic phenotype. PLAU+ Mac showed high expression of M1-related genes, and displayed relatively high M1, chemokine, and MHC scores, with BPs enriched in regulation of cell activation, suggesting that it may represent inflammatory macrophage in AD. C1QA+ Mac was similar to FOLR2+ Mac, which exhibited high expression of C1QA and FOLR2, and relatively high TRM score and the lowest M1 and chemokine scores, indicating that is may also be the TRM in AD. CD36+ Mac highly expressed CD36, SPP1, and PDIA4, showed the highest proteinase score, and were associated with translation-related cellular functions. IFIT1+ Mac, namely interferon-induced macrophage, highly expressed interferon-induced genes including IFIT1, IFIT3, and MX1, with enriched BPs and pathways related to defense response to virus. MGP+ Mac differentially expressed SMC- or fibroblast-related genes such as DCN, MGP, and TAGLN, suggesting it may play a role in aortic tissue remodeling.

##### 3.1.3.2 Other cells

Monocyte was characterized by FCN1, S100A12, and SELL, with BPs and pathways enriched in defense response and positive regulation of NF-κB transcription factor activity. Conventional DC (cDC) was marked by CD1C, CD1E, and FCER1A, and exhibited the highest MHC score, functioning cytoplasmic translation and peptide antigen assembly.

##### 3.1.3.3 Exploration on AD-enriched glycolysis-dominant ANGPTL4+ Mac

**Figure 4:**
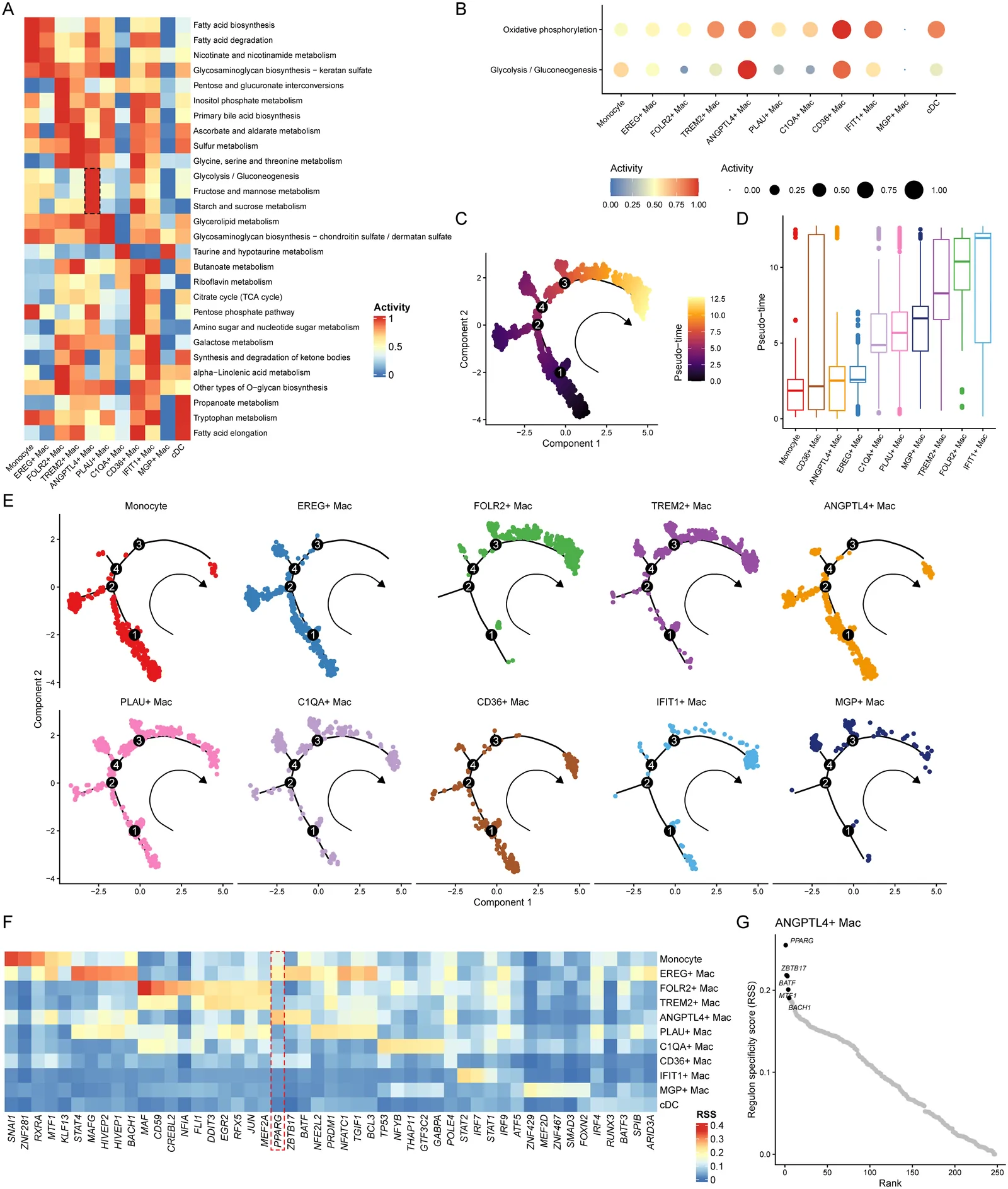
Further analyses on myeloid cells. **(A)** Activity heatmap of the top three metabolic pathway in each subpopulation. **(B)** Comparison of glycolysis/gluconeogenesis and oxidative phosphorylation pathway activities across all subpopulations. **(C)** Pseudo-time trajectory of monocytes and macrophages, with cell development from left to right. **(D)** Pseudo-time of each subpopulation, ranked in ascending order by average. **(E)** Distribution of each subpopulation along the pseudo-time trajectory. **(F)** RSS heatmap of the top five TFs for each subpopulation. **(G)** Top TFs regulating ANGPTL4+ Mac, ranked in descending order by RSS. RSS, regulon specificity score; TF, transcription factor.

We focused on investigating ANGPTL4+ Mac, a glycolytic phenotype macrophage enriched in AD. The boxplots of donor-level and dataset-level quantification of ANGPTL4+ Mac abundance were shown in **Supplementary Figures 2D–G**. The abundance of ANGPTL4+ Mac in AD was significantly higher than that in normal (P = 0.001), and no remarkable differences were observed across samples (P > 0.05). We calculated *R*_o/e_ value to compared the observed and expected cell numbers in each cluster. The results showed that ANGPTL4+ Mac was enriched in AD (*R*_o/e_ = 1.8), while it was not enriched in normal (*R*_o/e_ = 0.16) (**Supplementary Figure 2H**). We also calculated the entropy value of each cluster, finding that ANGPTL4+ Mac was not sample-specific or dataset-specific, but disease-specific (**Supplementary Figure 2I**). The metabolic pathway activity scores of all myeloid cells were calculated. ANGPTL4+ Mac demonstrated the highest activity in carbohydrate metabolic pathways like glycolysis/gluconeogenesis, fructose and mannose metabolism, and starch and sucrose metabolism among all pathways, and showed much higher activity compared to other myeloid cells (**Figure 4A**). Moreover, glycolysis/gluconeogenesis activity in ANGPTL4+ Mac was higher than oxidative phosphorylation, suggesting metabolic reprogramming under hypoxic conditions (**Figure 4B**). Moreover, ANGPTL4+ Mac had the highest glycolysis score in almost all methods (**Supplementary Figure 3A**). And the high expression level of glycolysis-related genes in ANGPTL4+ Mac also confirmed this (**Supplementary Figure 3B**). Pseudo-time trajectory analysis of monocytes and macrophages revealed that monocyte, ANGPTL4+ Mac, and EREG+ Mac were located at the initiation of the trajectory; C1QA+ Mac, PLAU+ Mac, and MGP+ Mac were positioned in the middle; and TREM2+ Mac, FOLR2+ Mac, and IFIT1+ Mac were situated at the end; while CD36+ Mac distributed throughout almost the entire trajectory (**Figures 4C–E**). Notably, the pseudo-time trajectory of ANGPTL4+ Mac resembles that of monocyte. Correlation and clustering analysis revealed that ANGPTL4+ Mac was strongly relevant to monocyte, and they could be clustered together, further supporting the similarity between them (**Supplementary Figure 3C**). We also identified genes whose expression significantly altered along the pseudo-time route and classified them into four clusters. Among these genes, ANGPTL4 first up-regulated, then down-regulated in early trajectory, and ultimately returning to a zero level (**Supplementary Figures 3D, E**). We used slingshot analysis to construct the cell lineages and confirmed the lineage direction from monocytes, EREG+/ANGPTL4+ Mac to other macrophages (**Supplementary Figures 3F–H**). We speculated that ANGPTL4 may represent an early monocyte-derived macrophage state, functioning within the monocyte/macrophage microenvironment without participating in subsequent molecular processes. TF regulatory network analysis indicated that, according to computed regulon specificity score (RSS) of identified TFs, ANGPTL4+ Mac may be regulated by PPARG, a top TF of ANGPTL4+ Mac, localizing together (**Figures 4F, G**; **Supplementary Figure 3I**).

#### 3.1.4 Identification of lymphocytes

We extracted primary T and B cells for secondary dimensionality reduction and clustering (dims = 1:10, res = 0.3), which yielded 10 cell clusters. These were ultimately identified as 4 categories comprising 10 cell types via DEGs and GSEA results of each individual cluster, including 5 clusters of T cells, 2 clusters of NK cells, 2 clusters of B cells, and 1 cluster of DCs (**Supplementary Figures 4A–E**). The cell proportion of GIMAP7+ T was elevated in AD (**Supplementary Figure 4F**).

##### 3.1.4.1 T and NK cells

CD4+ T highly expressed CD4, IL7R, and LTB, with BPs and pathways enriched in cytoplasmic translation and immune system development. CD8A+ T was identified by expression of CD8A, GZMK, and CRTAM, and enrichment in T cell receptor signaling pathway and T cell differentiation. GIMAP7+ T expressed GTPase of the immunity-associated protein family genes (10), such as GIMAP7 and GIMAP4, and oxidative phosphorylation and ATP synthesis are its main cellular processes. MGP+ T highly expressed contraction-related genes including MGP, TAGLN, and TPM2. The last T cell cluster, designated JUN+ T, highly expressed stress-related genes like JUN and FOS, with enriched BPs and pathways of chromatin remodeling and positive regulation of RNA metabolic process. NCAM1+ NK (CD56^bright^ NK) highly expressed NCAM1, KLRC1, and XCL1, and FCGR3A+ NK (CD56^dim^ NK) was characterized by expression of GZMB, KLRD1, and FCGR3A. Both the two clusters of NK cells functioned in NK cell mediated cytotoxicity.

##### 3.1.4.2 B, plasma and other cells

B cell highly expressed CD79A, HLA-DQA1, and CD22, with BPs and pathways enriched in peptide antigen assembly. Plasma cell highly expressed MZB1, JCHAIN, and IGKC, and its BPs were enriched in glycosylation and B cell mediated immunity. Plasmacytoid DC (pDC) was characterized by high expression of CLEC4C and IL3RA and cellular functions of antigen processing and presentation. Although pDC is of myeloid origin, it was initially clustered within B cell due to their shared characteristics and was identified upon secondary clustering.

#### 3.1.5 Cell-cell communication across whole identified cells

**Figure 5:**
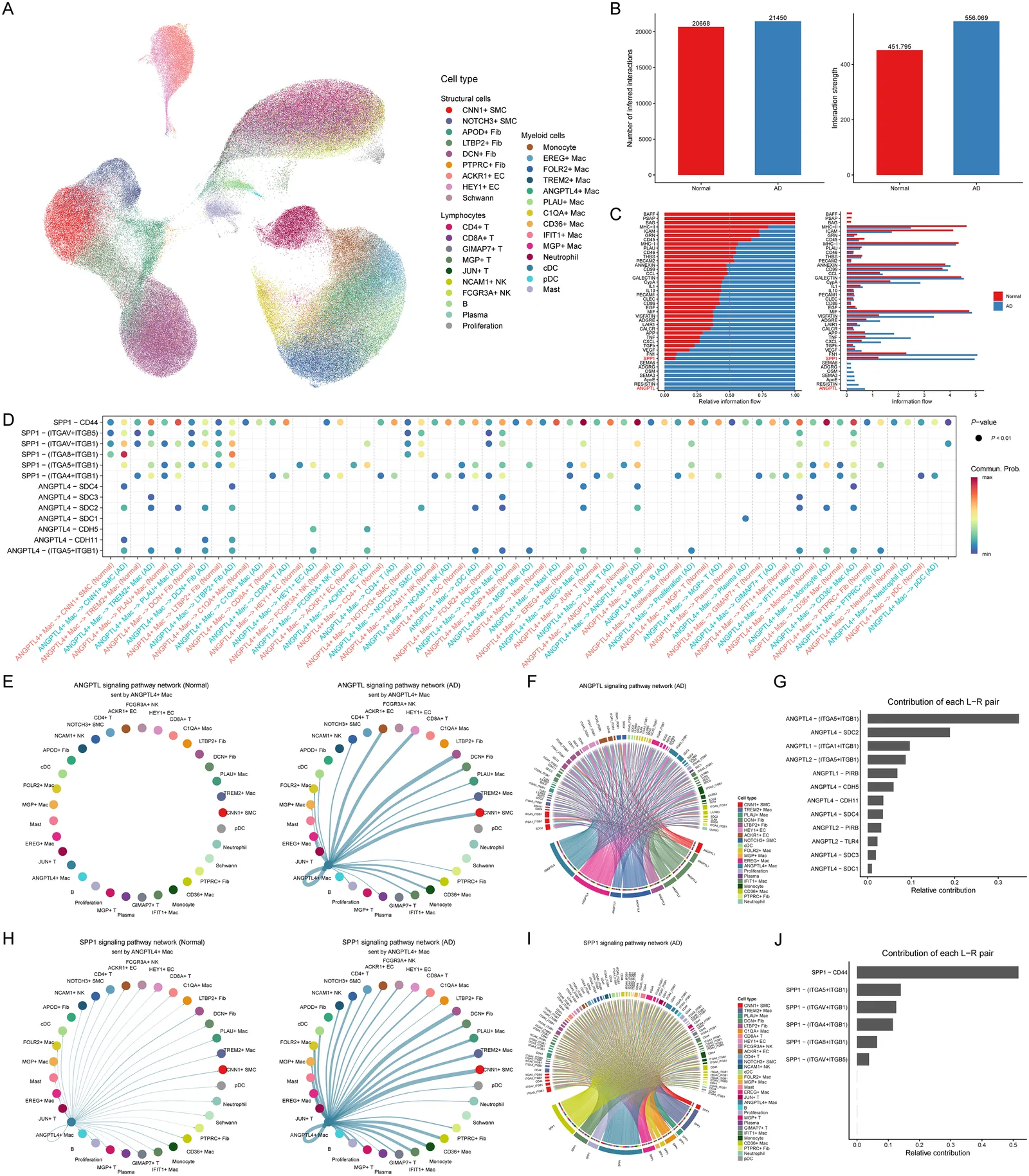
Cell-cell communication across all identified cell subpopulations. **(A)** UMAP plot of all identified cell subpopulations. **(B)** Comparison of the number and strength of cell-cell interactions between normal and AD samples. **(C)** Cell-cell communication signaling pathways involving ANGPTL4+ Mac. **(D)** Dot plot of cell-cell communications mediated by ANGPTL4+ Mac through ANGPTL and SPP1 signaling pathways. **(E)** ANGPTL signaling pathway network mediated by ANGPTL4+ Mac as signal sender in normal and AD samples. **(F)** Chord diagram of ANGPTL signaling pathway in AD. **(G)** Ligand-receptor pairs contributing to ANGPTL signaling pathway in AD. **(H)** SPP1 signaling pathway network mediated by ANGPTL4+ Mac as signal sender in normal and AD samples. **(I)** Chord diagram of SPP1 signaling pathway in AD. **(J)** Ligand-receptor pairs contributing to SPP1 signaling pathway in AD. UMAP, uniform manifold approximation and projection; AD, aortic dissection.

The integrated UMAP plot of entire cell subpopulations was shown in **Figure 5A**. We quantified and compared the interaction strength across all identified cell subpopulations between normal and AD samples. Cell-cell communications were considerably enhanced in AD (**Figure 5B**; **Supplementary Figure 5A**). Focusing on ANGPTL4+ Mac, ANGPTL pathway exhibited the strongest specificity to AD in terms of signaling pathways, and SPP1 pathway demonstrated the most pronounced intensification in signaling strength in AD (**Figure 5C**). ANGPTL4+ Mac participated in ANGPTL and SPP1 signaling pathways through ANGPTL4- and SPP1-related ligand-receptor pairs, chatting with multiple cells and exhibiting distinctions between normal and AD (**Figure 5D**). The communication intensity of ANGPTL pathway was higher in AD, and when ANGPTL4+ Mac served as signal sender, this pathway was exclusively present in AD (**Figure 5E**; **Supplementary Figure 5B**). In AD, ANGPTL signaling pathway was primarily mediated by ANGPTL4-(ITGA5/ITGB1) ligand-receptor pair, occurring between ANGPTL4+ Mac and LTBP2+ Fib or CNN1+ SMC (**Figures 5F, G**; **Supplementary Figures 5C, D**). Similar to the ANGPTL signaling pathway, the communication intensity of SPP1 signaling pathway was also excessive in AD, with a particularly evident enhancement when ANGPTL4+ Mac acted as signal sender (**Figure 5H**; **Supplementary Figure 5E**). In AD, SPP1 signaling pathway was principally mediated by SPP1-CD44 ligand-receptor pair, occurring between ANGPTL4+ Mac and LTBP2+ Fib or CNN1+ SMC (**Figures 5I, J**; **Supplementary Figures 5F, G**). These results suggest that in AD, cell-cell communications were more extensive, and among the remarkably enhanced communications, ANGPTL4+ Mac showed inferred ligand-receptor interactions with LTBP2+ Fib or CNN1+ SMC via ANGPTL4-(ITGA5/ITGB1) and SPP1-CD44 axes.

### 3.2 Linking ANGPTL4+ Mac with bulk RNA-seq

#### 3.2.1 Integration of bulk RNA-seq datasets

**Figure 6:**
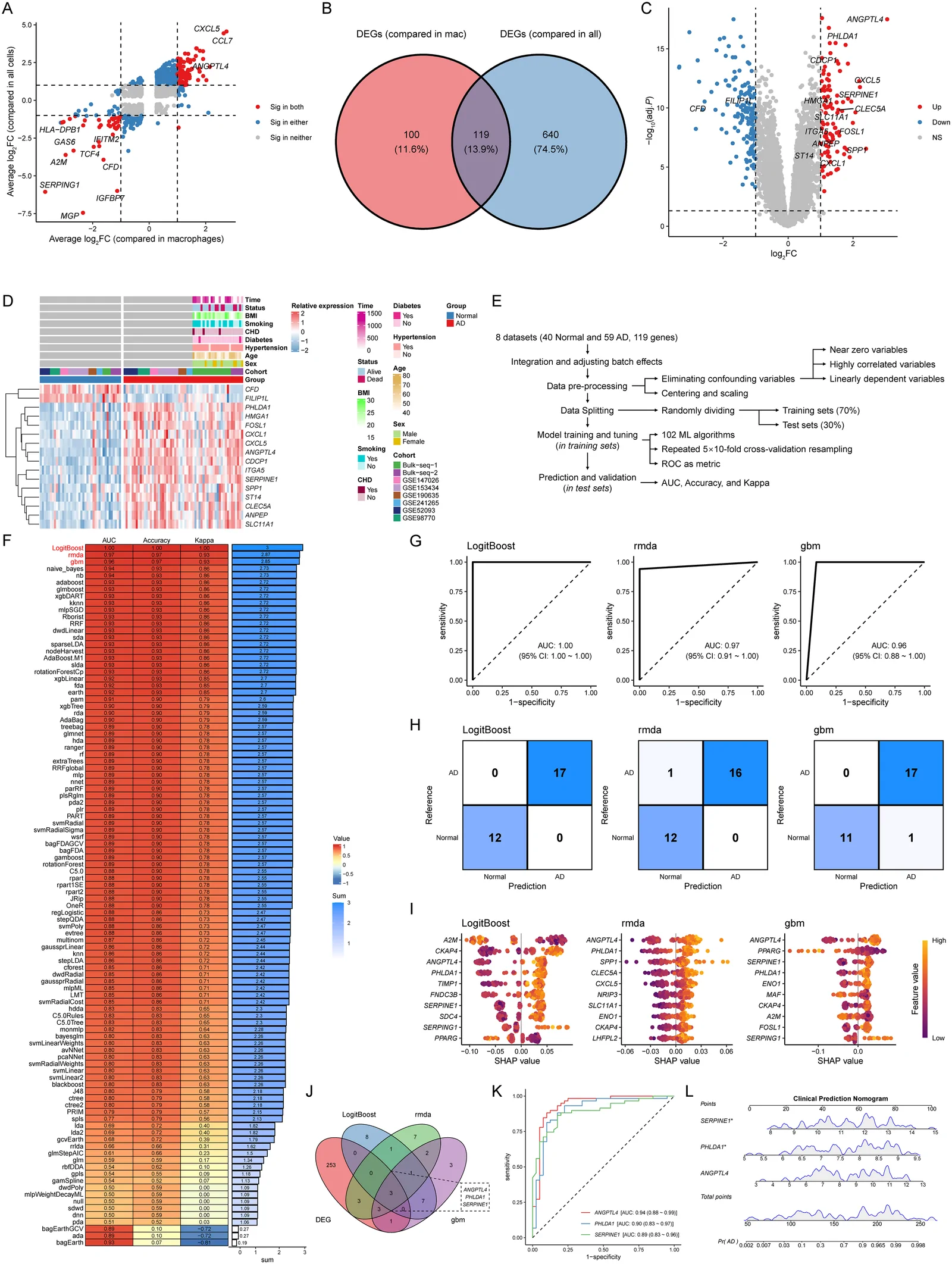
Screening of signature genes and construction of clinical prediction model. **(A)** DEGs of ANGPTL4+ Mac compared in myeloid cells and compared in all cells. **(B)** Venn diagram of DEGs of ANGPTL4+ Mac. **(C)** Volcano plot of DEGs in integrated bulk RNA-seq data. **(D)** Heatmap of the expression profiles of DEGs of ANGPTL4+ Mac. **(E)** The workflow of ML. **(F)** Summary of model performance for 102 ML algorithms fitted on test set. **(G)** ROC curves of the top three best-performing ML models on test set. **(H)** Confusion matrices of the top three best-performing ML models on test set. **(I)** SHAP value beeswarm plots for the top 10 genes with the greatest contribution to each of the top three ML algorithms. **(J)** Venn diagram of the top 20 genes with the greatest contribution to model performance across the top three algorithms. **(K)** ROC curves for the three identified signatures across all samples. **(L)** Nomogram of multivariate logistic regression model constructed based on the three identified signatures. DEG, differentially expressed gene; ML, machine learning; ROC, receiver operating characteristic; SHAP, Shapley additive explanations.

To investigate the potential molecular mechanisms of ANGPTL4+ Mac, we identified and overlapped DEGs in ANGPTL4+ Mac compared to other myeloid cells and to all other cells (|avg_log2FC| > 1, p_val_adj < 0.05, and pct > 0.25), yielding 119 intersection genes (**Figures 6A, B**). We retrieved these genes from the STRING database and then constructed and visualized the protein-protein interaction (PPI) network (**Supplementary Figure 6A**). We acquired and enrolled six public bulk RNA-seq datasets of TAAD, which were integrated with two in-house batches of our cohorts followed by batch effect adjustment (**Supplementary Figures 6B–E**). The integrated bulk RNA-seq data comprised 99 samples with expression of 12,746 genes, including 40 normal and 59 AD samples. Differential expression analysis identified 99 up-regulated and 164 down-regulated DEGs (**Figure 6C**). Among these, 14 were significantly up-regulated and 2 were significantly down-regulated in the bulk RNA-seq data (**Figure 6D**). Notably, ANGPTL4 showed the most remarkable up-regulation (logFC = 3.05, adj.P.Val = 3.22×10^-18^).

#### 3.2.2 Screening signatures using machine learning algorithms

Our ML workflow is illustrated in **Figure 6E**. After pre-processing the gene expression matrix, a total of 99 variables were included for ML modeling. All algorithms were first tuned in the training set using a random parameter grid, with ROC as the evaluation metric to fit the optimal model. The fitted models were then used to predict sample group in the test set and were compared and evaluated against real-world observations. Finally, the best ML algorithms were selected based on the AUC, Accuracy, and Kappa values obtained from results on fitting test set. Among the 102 classification ML algorithms involved, Boosted Logistic Regression (LogitBoost), Robust Mixture Discriminant Analysis (rmda), and Stochastic Gradient Boosting (gbm) evinced the best predictive performance and optimal model fitting, achieving AUC values above 0.95 (**Figures 6F–H**; **Supplementary Table 3**). We also conducted leave-one-cohort-out validation to validate the best three ML algorithms, and their average AUC was over 0.90 (**Supplementary Figure 6F**). After intersecting the top 20 genes with the greatest contribution to model performance across the three algorithms, we obtained three signature genes including ANGPTL4, PHLDA1, and SERPINE1. These three signatures consistently made important contributions across the three optimal models, all exerting positive effects on AD (**Figures 6I, J**). The ROC curves for the three signatures across all samples demonstrated AUC values above 0.89, indicating satisfied performance in distinguishing between normal people and AD patients (**Figure 6K**).

#### 3.2.3 Construction and assessment of clinical prediction model

A multivariate logistic model was constructed based on the three signatures, and a nomogram was then depicted (**Figure 6L**). A C-index of 0.95 (95% CI: 0.91 – 0.99) and a P-value of 0.5 from Hosmer-Lemeshow test indicated consistency between model predictions and actual observations, which was further validated by the calibration curve (**Supplementary Figure 6G**). The clinical decision curve analysis (DCA) implied that, regardless of whether clinical decisions tend to be aggressive (low threshold) or conservative (high threshold), using this model to guide clinical decision-making may achieve a relatively high net clinical benefit (**Supplementary Figure 6H**). Moreover, compared with single biomarkers, this model provided a greater net benefit. However, due to the lack of an external cohort, our prediction model remains to be further validated.

#### 3.2.4 Deconvolution-derived cell compositions in TAAD

**Figure 7:**
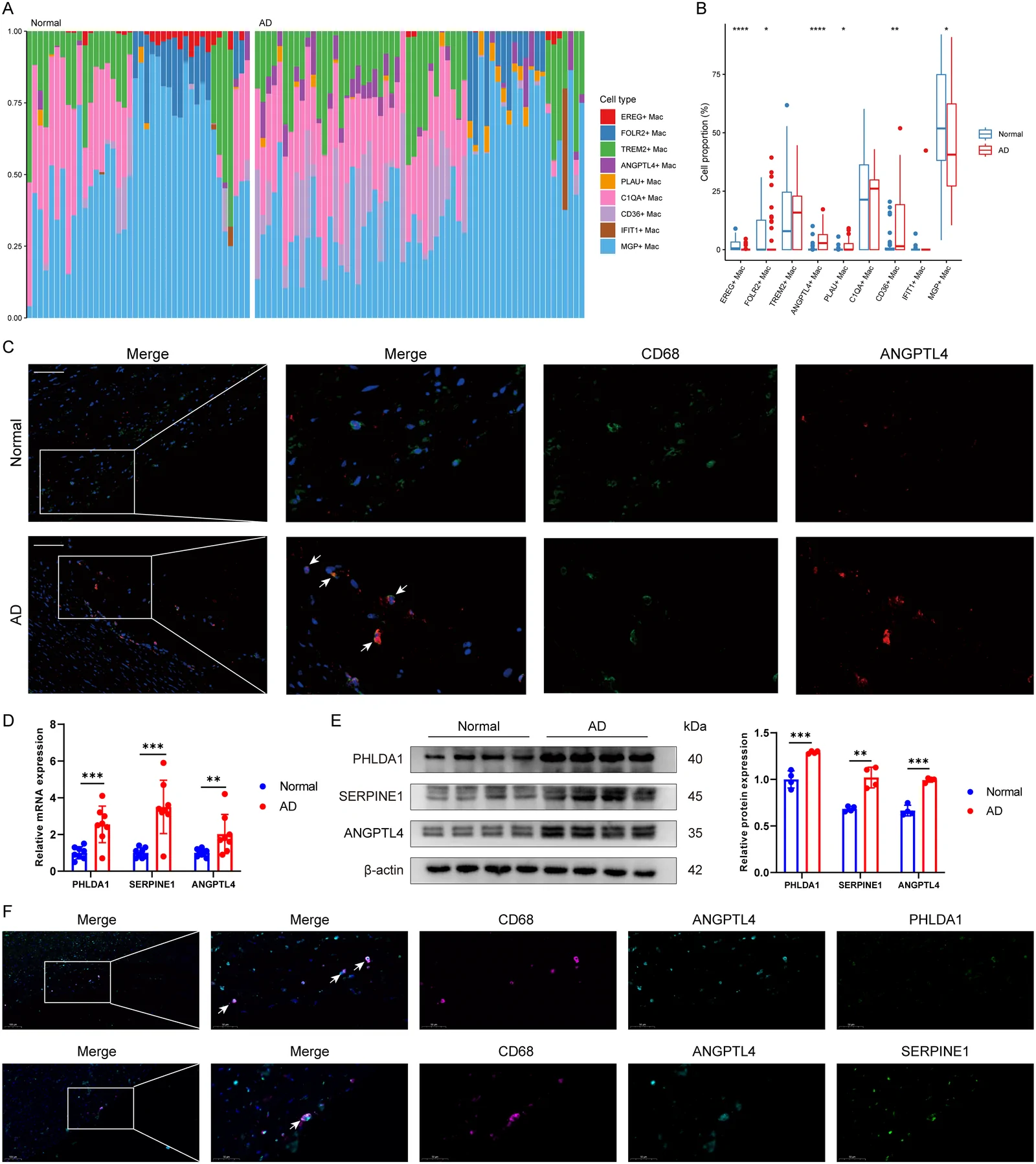
Clinical relevance of deconvolution-derived macrophage subpopulation proportions and *in vitro* experimental validation. **(A)** The proportions of deconvoluted macrophage subpopulations in each bulk sample. **(B)** Differences in inferred macrophage proportions between normal and AD samples. **(C)** Zoom-in immunofluorescence images of ANGPTL4+ Mac identified by co-localization of CD68 and ANGPTL4. Scale bars = 100 μm. White arrows indicate ANGPTL4+ Mac. **(D)** Results of qRT-PCR of signature gene mRNA in AD. **(E)** WB of signature proteins in AD. **(F)** Immunofluorescence co-localization of CD68, ANGPTL4, PHLDA1, and SERPINE1. *P < 0.05, **P < 0.01, ***P < 0.001, ****P < 0.0001. bulk RNA-seq, bulk RNA sequencing; scRNA-seq, single-cell RNA sequencing; AD, aortic dissection; qRT-PCR, quantitative real-time polymerase chain reaction; WB, western blot.

We performed deconvolution on bulk RNA-seq (n = 99) using annotated scRNA-seq (n = 39) to estimate the proportions of cell subpopulations in each bulk sample. The results showed that in AD, the proportion of EREG+ Mac was significantly decreased, while ANGPTL4+ Mac was significantly increased (P < 0.0001) (**Figures 7A, B**; **Supplementary Table 2**). We also conducted a sensitivity analysis using leave-one-dataset-out reference and re-estimated the proportion of macrophage subpopulations in integrated bulk RNA-seq data. The results showed that ANGPTL4+ macrophage was still significantly enriched in AD (**Supplementary Figure 6I**). ROC curves for each macrophage subpopulation revealed that only EREG+ Mac and ANGPTL4+ Mac reached an AUC greater than 0.75, suggesting that ANGPTL4+ Mac had a certain diagnostic value for AD (**Supplementary Figure 6J**). The ssGSEA results indicated that the glycolysis score was significantly higher in AD samples, suggesting enhanced glycolysis metabolism in AD (**Supplementary Figure 6K**). Correlation analysis among the three signatures, macrophage subpopulation compositions, and glycolysis scores showed that ANGPTL4+ Mac exhibited a significant positive correlation with signatures and glycolysis score (**Supplementary Figures 6L–N**).

#### 3.2.5 Exploratory clinical relevance analysis of deconvoluted macrophage

We collected clinical profiles from AD patients (n = 25) in the in-house sequencing cohort to explore the clinical relevance of the cell compositions in AD. Among these, ANGPTL4+ Mac showed a suggestive significant positive correlation with monocyte count and creatinine, and negative correlation with fibrinogen (**Supplementary Figure 6O**; **Supplementary Table 4**). The positive correlation between ANGPTL4+ Mac and monocyte count is consistent with our pseudo-time analysis inference. However, our exploratory analysis was executed in a small cohort, and future verification is needed in a larger cohort.

### 3.3 Experimental validation of ANGPTL4+ Mac in AD

Immunofluorescence staining was performed on both normal and dissected aortic tissues. Co-localization of CD68 and ANGPTL4 indicated the presence of ANGPTL4+ Mac. While ANGPTL4+ Mac was barely detectable in normal tissues, CD68+ ANGPTL4+ cells were obviously observed in AD tissues (**Figure 7C**). This finding is consistent with our bioinformatics analyses, which identified ANGPTL4+ Mac as an AD-enriched macrophage subpopulation. Furthermore, qRT-PCR and WB of aortic tissues both confirmed significant up-regulation of the three signatures in AD (**Figures 7D, E**), and PHLDA1 and SERPINE1 were confirmed to be co-localized with ANGPTL4+ Mac (**Figure 7F**), highlighting the important role of ANGPTL4+ Mac and its signatures in the pathogenesis of AD.

## 4 Discussion

In this study, we developed a comprehensive single-cell atlas of TAAD by integrating six publicly available scRNA-seq datasets, providing a high-resolution depiction of cellular heterogeneity within the dissected human aorta. Through systematic cell annotation, we identified a macrophage subpopulation enriched in AD, termed ANGPTL4+ Mac. This subpopulation was predominantly enriched in AD tissues, exhibited a unique glycolytic metabolic profile, represented an early monocyte-derived macrophage state, and appeared to be potentially regulated by PPARG. Analysis of cell-cell communication indicated that ANGPTL4+ Mac showed inferred ligand-receptor interactions with structural cells through ANGPTL and SPP1 signaling pathways. By amalgamating bulk RNA-seq datasets from both public and our in-house cohorts, and employing ML algorithms, we identified ANGPTL4, PHLDA1, and SERPINE1 as core markers of this macrophage subset. Further deconvolution and correlation analysis associated the proportion of ANGPTL4+ Mac with glycolysis scores and clinical parameters, such as monocyte count. Experimental validation demonstrated an increase in ANGPTL4+ Mac along with elevated expression levels of ANGPTL4, PHLDA1, and SERPINE1 in AD. These findings collectively reveal an AD-enriched macrophage subset, which may hold pronounced therapeutic potential.

The most noteworthy finding of our study is the identification of ANGPTL4+ Mac as an enriched subset in AD. ANGPTL4 is a secreted glycoprotein that plays a role in lipid metabolism, angiogenesis, and inflammation, participating in cardiovascular diseases (CVDs) including atherosclerosis and heart failure (52–54). However, its involvement in AD and association with a distinct macrophage subset have not been documented. Our single-cell analysis demonstrated that ANGPTL4+ Mac exhibited elevated glycolytic activity and were enriched in hypoxia and glycolysis pathways, indicating metabolic reprogramming within the local aortic microenvironment. These findings are consistent with the hypoxic conditions typically observed in the diseased aortic wall and aligns with the emerging concept that metabolic shifts influence macrophage polarization and function (55–57). Importantly, pseudo-time trajectory analysis revealed that ANGPTL4+ Mac was positioned early in the differentiation trajectory, closely related to monocytes. This suggests that they may represent an early-stage monocyte-derived macrophage subset in the dissected aorta. The positive association between ANGPTL4+ Mac proportion and monocyte count in our in-house cohort reinforces this idea. SCENIC analysis highlighted PPARG may be a major TF for ANGPTL4+ Mac. As a master regulator of macrophage polarization and metabolism, the activation of PPARG is associated with glycolytic changes in low-oxygen conditions, making it a potential therapeutic target for influencing ANGPTL4+ Mac behavior (57, 58).

Our integrative single-cell atlas identified several features enriched in AD. Firstly, a significant increase in the proportion of LTBP2+ Fib (a subset of fibromyocyte) in AD, which algorithmically aligns with the concept of SMC phenotypic switching (4). Secondly, within the myeloid cell subpopulation, ANGPTL4+ Mac was predominantly observed in AD, whereas FOLR2+ Mac (a subset of TRM) was more prevalent in normal aortas. This distinct cellular composition indicates that the immune landscape in AD is biased toward an inflammatory and metabolically reprogrammed state, as opposed to a homeostatic state (59). Cell-cell communication analysis demonstrated that ANGPTL4+ Mac mainly interacted with LTBP2+ Fib and CNN1+ SMC through ANGPTL4-(ITGA5/ITGB1) and SPP1-CD44 pairs. These interactions were much stronger in AD, indicating that ANGPTL4+ Mac may play a vital role in pathological interactions between immune and structural cells. SPP1 is known for its pro-inflammatory and matrix-remodeling roles in CVDs (60), which modulate macrophage behavior and SMC phenotypic switching (61, 62). The simultaneous activation of ANGPTL and SPP1 signaling pathways implies that ANGPTL4+ Mac might create a microenvironment that promotes AD progression. Further research with co-culture systems or conditional knockout models is needed to explore the roles of these interactions.

Utilizing ML algorithms applied to combined bulk RNA-seq data, we identified ANGPTL4, PHLDA1, and SERPINE1 as the principal signatures. Remarkably, each of these genes has been implicated in cardiovascular pathology. ANGPTL4 facilitates SMC phenotypic switching and dysfunctions in AD (63). PHLDA1 plays a role in oxidative stress-induced cardiomyocyte injury and myocardial ischemia reperfusion injury (64). SERPINE1 serves as a critical regulator of fibrinolysis and ECM turnover, with increased expression linked to adverse vascular remodeling (65, 66). The identification of these signatures as primary contributors to ML models highlights their potential as biomarkers for AD. Furthermore, a logistic regression model incorporating the three signatures demonstrated well diagnostic performance and clinical utility, indicating that a combinatorial biomarker approach may surpass the efficacy of single markers in distinguishing AD patients from healthy individuals. Through the deconvolution of bulk RNA-seq data utilizing the integrated scRNA-seq reference, we identified a significant increase of ANGPTL4+ Mac in AD. Notably, glycolysis scores exhibited positive correlations with the ANGPTL4+ Mac proportion and the three signatures, suggesting that modulating glycolysis in ANGPTL4+ Mac could be investigated as a potential therapeutic approach for AD.

Clinical correlation analysis revealed that the proportion of ANGPTL4+ Mac positively correlated with monocyte count and creatinine levels, while negatively correlated with fibrinogen levels. AD is an inflammatory condition, and elevated peripheral blood monocyte count is a common manifestation of systemic inflammatory response (67). Moreover, the local tear of dissected aorta requires substantial recruitment of monocytes that differentiate into macrophages to participate in tissue remodeling (7). Meanwhile, a multicenter study in China suggested that elevated monocyte count is associated with poor postoperative prognosis in AD (68). AD often involves the renal artery or leads to malperfusion, subsequently triggering acute kidney injury and elevated serum creatinine levels (69). The preoperative serum creatinine levels could assist in predicting outcomes in patients undergoing surgery for AD (70). We suppose that the correlation between ANGPTL4+ Mac and creatinine levels may reflect the systemic inflammatory burden or renal involvement in AD. The negative correlation between the proportion of ANGPTL4+ Mac and fibrinogen suggested that a higher proportion of ANGPTL4+ Mac may imply a more severe local or systemic coagulation/fibrinolysis imbalance, leading to more fibrinogen consumption. Meanwhile, the persistent depletion of fibrinogen during the progression of AD disrupts vascular stability, leading to phenotypic switching of SMCs and impairment of the integrity of aortic structure (71).

This study has several limitations. First, despite integrating multiple scRNA-seq datasets to enhance statistical power, the sample size remained limited, and we lacked spatial transcriptomic sequencing as well as scRNA-seq of peripheral blood to corroborate our findings. Second, although we performed quality control and corrected batch effects on the scRNA-seq data, these procedures may still have influenced our results. Third, owing to the low incidence of aortic dissection, our exploratory clinical correlation analysis was conducted on a small single-center cohort, necessitating larger-scale, prospective, multicenter studies to validate the diagnostic and therapeutic potential of ANGPTL4+ Mac and the three identified signatures. Fourth, our findings warrant further validation and investigation through additional experiments, such as flow cytometry and animal experiments. Fifth, this study primarily relied on static transcriptomic data, which overlook the dynamic changes during the progression and repair of AD. Future investigations should focus on longitudinal sampling and sequencing to capture the molecular and cellular dynamics.

## 5 Conclusions

In summary, our study using single-cell and bulk transcriptomics, along with ML and experiments, identified a unique AD-enriched ANGPTL4+ Mac subset with glycolytic phenotype, which represented an early monocyte-derived macrophage state, predicted to be regulated by PPARG and showed inferred ligand-receptor interactions with structural cells. The core signatures including ANGPTL4, PHLDA1, and SERPINE1 showed strong diagnostic efficacy and clinical relevance. These insights enhance our understanding of cellular and molecular landscape of AD and support the development of macrophage-targeted diagnosis and therapy strategies.

## Data Availability

Our bulk RNA-seq data and R codes presented in the study are deposited in the GitHub repository (https://github.com/CQMULZH/AD_scRNA_Integration).
