# Supplementary material for: Integration of single-cell and bulk RNA sequencing data identified an aortic dissection-enriched ANGPTL4+ macrophage subpopulation
Source: Front Immunol. 2026 Aug 28;17:1874233. doi: 10.3389/fimmu.2026.1874233 (PMC13561848; doi:10.3389/fimmu.2026.1874233)
Supplement: Supplementary file 1 [file Supplementaryfile1.pdf]

Supplementary Material

1 Supplementary Figures and Tables

1.1 Supplementary Figures

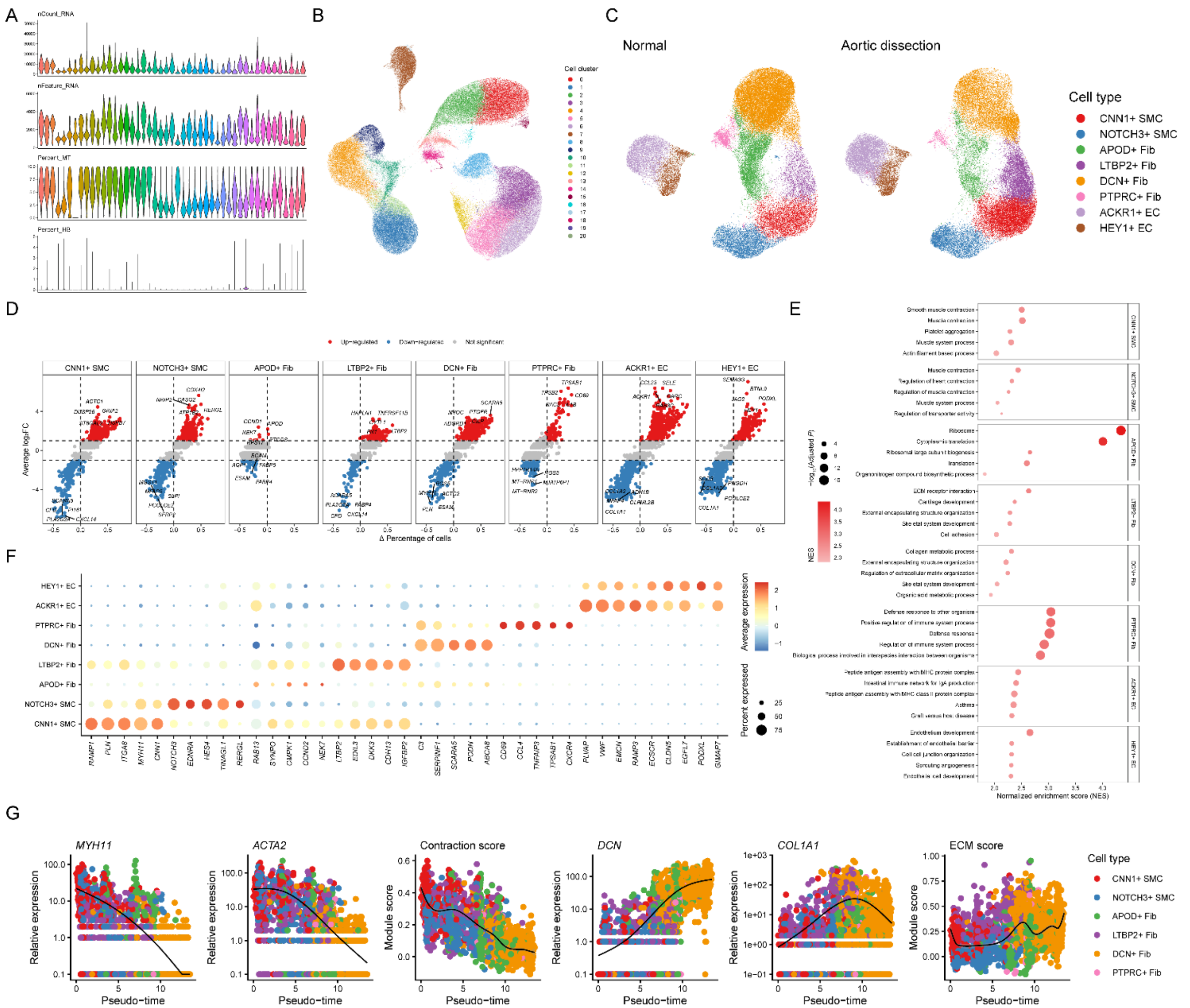

Supplementary Figure 1. Integration of scRNA-seq data and analysis of structural cells. (A) Quality control of the integrated scRNA-seq data. (B) Primary dimensionality reduction and clustering yielded 21 cell clusters. (C) UMAP plots of level 2 cell annotation for structural cells in normal and AD samples. (D) DEGs for each subpopulation. (E) Enriched BPs and pathways for each subpopulation. (F) Top 5 up-regulated DEGs for each structural cell subpopulation. (G) Changes in contraction-related genes and cell scores, as well as ECM-related genes and cell scores, along the pseudo-time trajectory. Abbreviations: scRNA-seq, single-cell RNA sequencing; UMAP, uniform manifold approximation and projection; AD, aortic dissection; DEG, differentially expressed gene;

ECM, extracellular matrix.

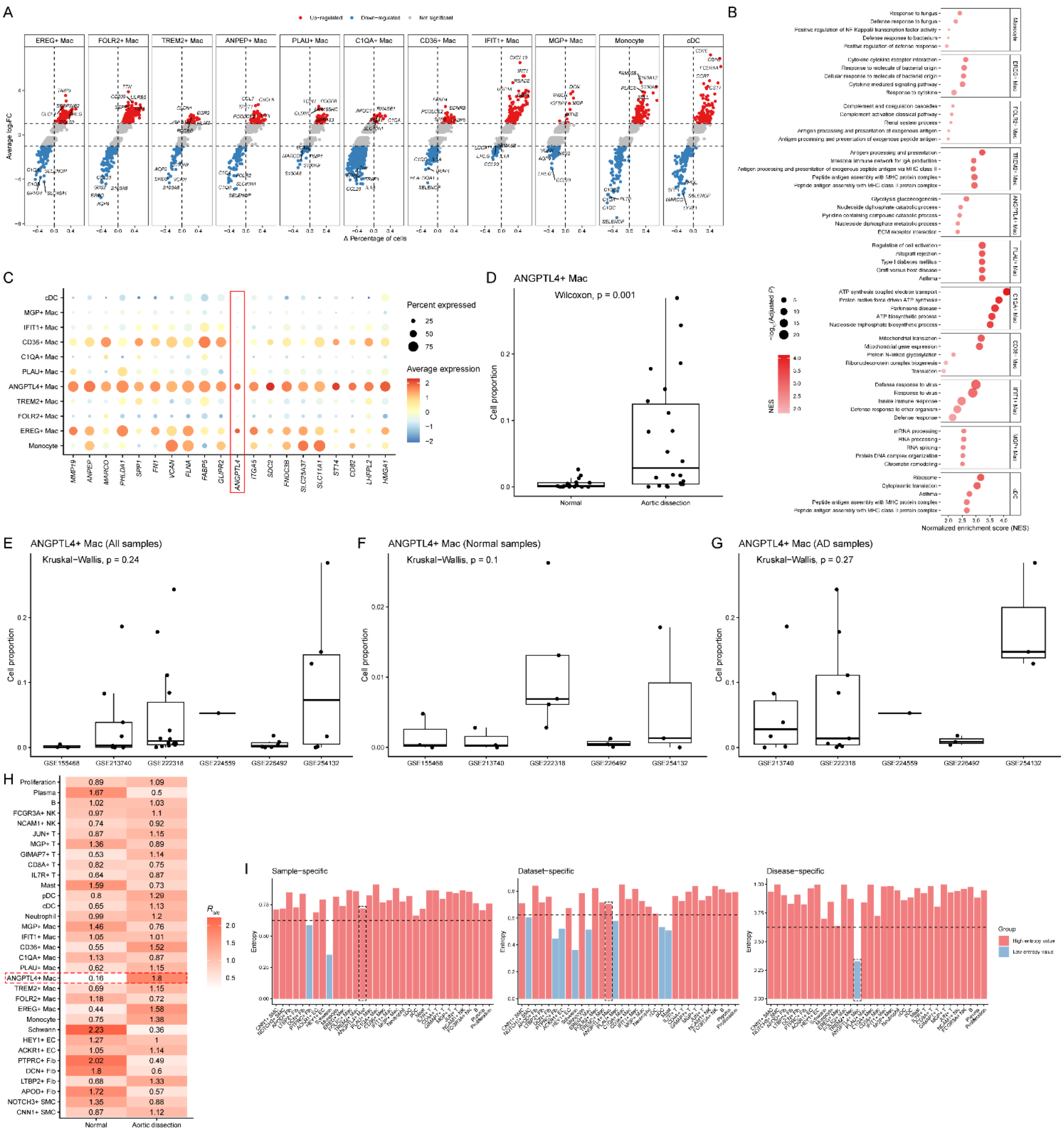

Supplementary Figure 2. Level 2 cell annotation and quantification of enrichment and specificity of myeloid cells. (A) DEGs for each subpopulation. (B) Enriched BPs and pathways for each subpopulation. (C) Expression profiles of the top 20 up-regulated DEGs in ANGPTL4+ Mac across all myeloid cells, in order to determine the naming of this myeloid cell subpopulation. (D) Boxplots of donor-level quantification of ANGPTL4+ Mac abundance. (E) Boxplots of dataset-level quantification of ANGPTL4+ Mac abundance in all samples. (F) Boxplots of dataset-level quantification of ANGPTL4+ Mac abundance in normal samples. (G) Boxplots of dataset-level

quantification of ANGPTL4+ Mac abundance in AD samples. (H) $R_{o/e}$ value of each cluster in different group. (I) Entropy value of each cluster in different conditions. Abbreviations: DEG, differentially expressed gene; BP, biological process; AD, aortic dissection.

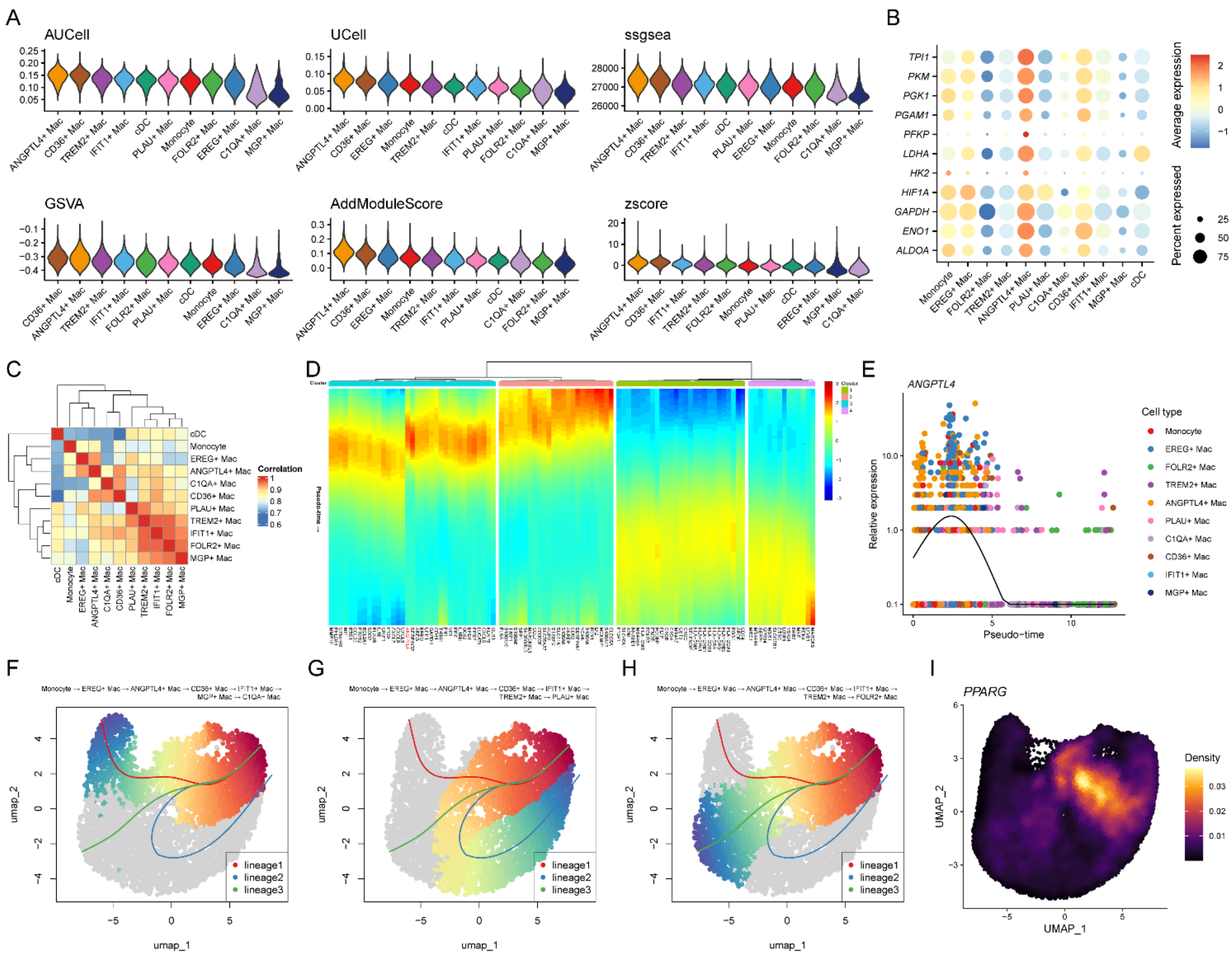

Supplementary Figure 3. Additional analyses of myeloid cells. (A) Glycolysis score of myeloid cells using six methods. (B) Expression of glycolysis-related genes in myeloid cells. (C) Correlation clustering heatmap of subpopulations. (D) Clustering heatmap of the top 100 genes with the most significant expression changes along pseudo-time. (E) Expression trend of ANGPTL4 along pseudo-time. (F) The first cell lineage of myeloid cells constructed by slingshot. (G) The second cell lineage of myeloid cells constructed by slingshot. (H) The third cell lineage of myeloid cells constructed by slingshot. (I) UMAP plot of PPARG expression density in myeloid cells. Abbreviation: UMAP, uniform manifold approximation and projection.

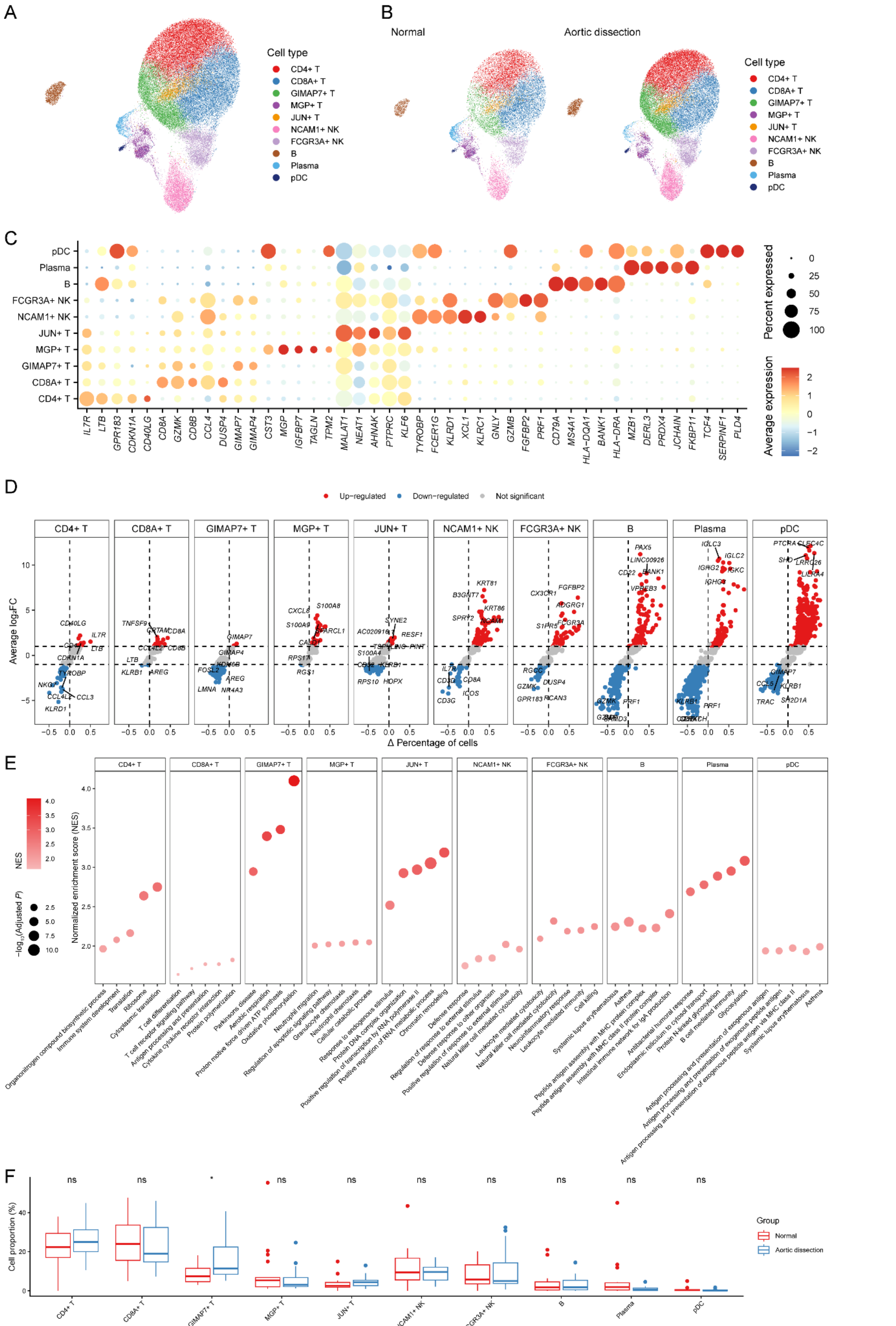
A
Cell type
CD4+ T
CD8A+ T
GIMAP7+ T
MGP+ T
JUN+ T
NCAM1+ NK
FCGR3A+ NK
B
Plasma
pDC
B
Normal
Aortic dissection
C
Percent expressed
0
25
50
75
100
Average expression
IL7R LTB GPR183 CDKN1A CD40LG CD8A GZMK CD8B CCL4 DUSP4 GIMAP7 GIMAP4 CST3 MGP IGFBP7 TAGLN TPM2 MALAT1 NEAT1 AHNAK PTPRC KLF6 TYROBP FCER1G KLRD1 XCL1 KLRC1 GNLY GZMB FGFBP2 PRF1 CD79A MS4A1 HLA-DQA1 BANK1 HLA-DRA MZB1 DERL3 PRDX4 JCHAIN FKBP11 TCF4 SERPINF1 PLD4
D
Up-regulated
Down-regulated
Not significant
Average log2FC
Δ Percentage of cells
E
NES
Normalized enrichment score (NES)
F
Cell proportion (%)
Group
Normal
Aortic dissection
ns
*

Supplementary Figure 4. Level 2 cell annotation of lymphocytes. (A) UMAP plot of level 2 cell annotated lymphocytes after secondary dimensionality reduction and clustering. (B) UMAP plots of level 2 cell annotations of lymphocytes in normal and AD samples. (C) Top 5 up-regulated DEGs for each lymphocyte subpopulation. (D) DEGs for each subpopulation. (E) Enriched BPs and pathways for each subpopulation. (F) Differences in proportions of each subpopulation between normal and AD samples (* P < 0.05). Abbreviations: UMAP, uniform manifold approximation and projection; AD, aortic dissection; DEG, differentially expressed gene; BP, biological process.

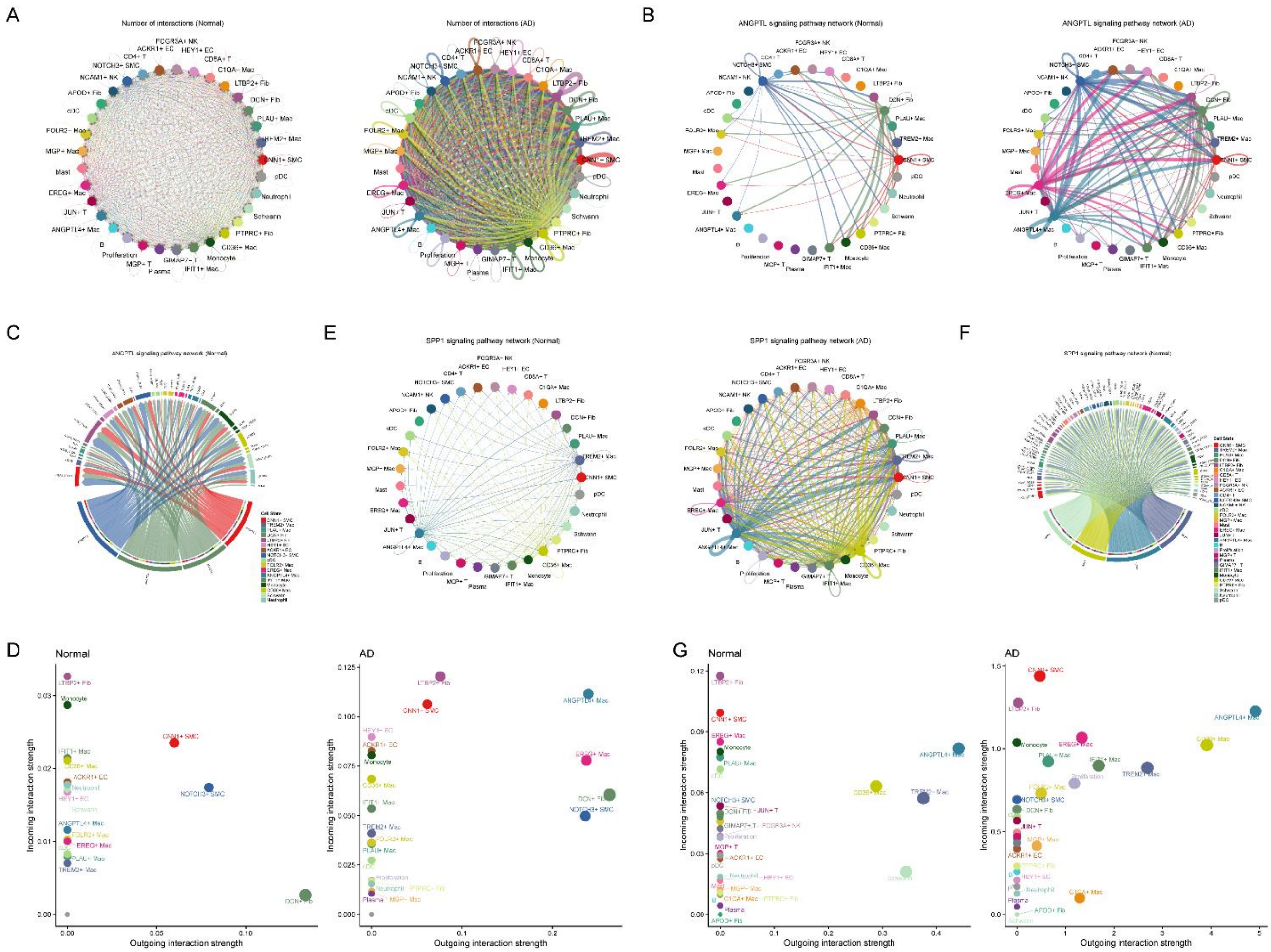

Supplementary Figure 5. Cell-cell communication analysis across all identified cell subpopulations. (A) Interaction networks of cell-cell communication counts in normal and AD samples. (B) ANGPTL signaling pathway network in normal and AD samples. (C) Chord diagram of ANGPTL signaling pathway in normal samples. (D) Incoming and outgoing interaction strength of ANGPTL signaling pathway across all identified cell subpopulations in normal and AD samples. (E) SPP1 signaling pathway network in normal and AD samples. (F) Chord diagram of SPP1 signaling pathway in normal samples. (G) Incoming and outgoing interaction strength of SPP1 signaling pathway across all identified cell subpopulations in normal and AD samples. Abbreviations: AD, aortic dissection.

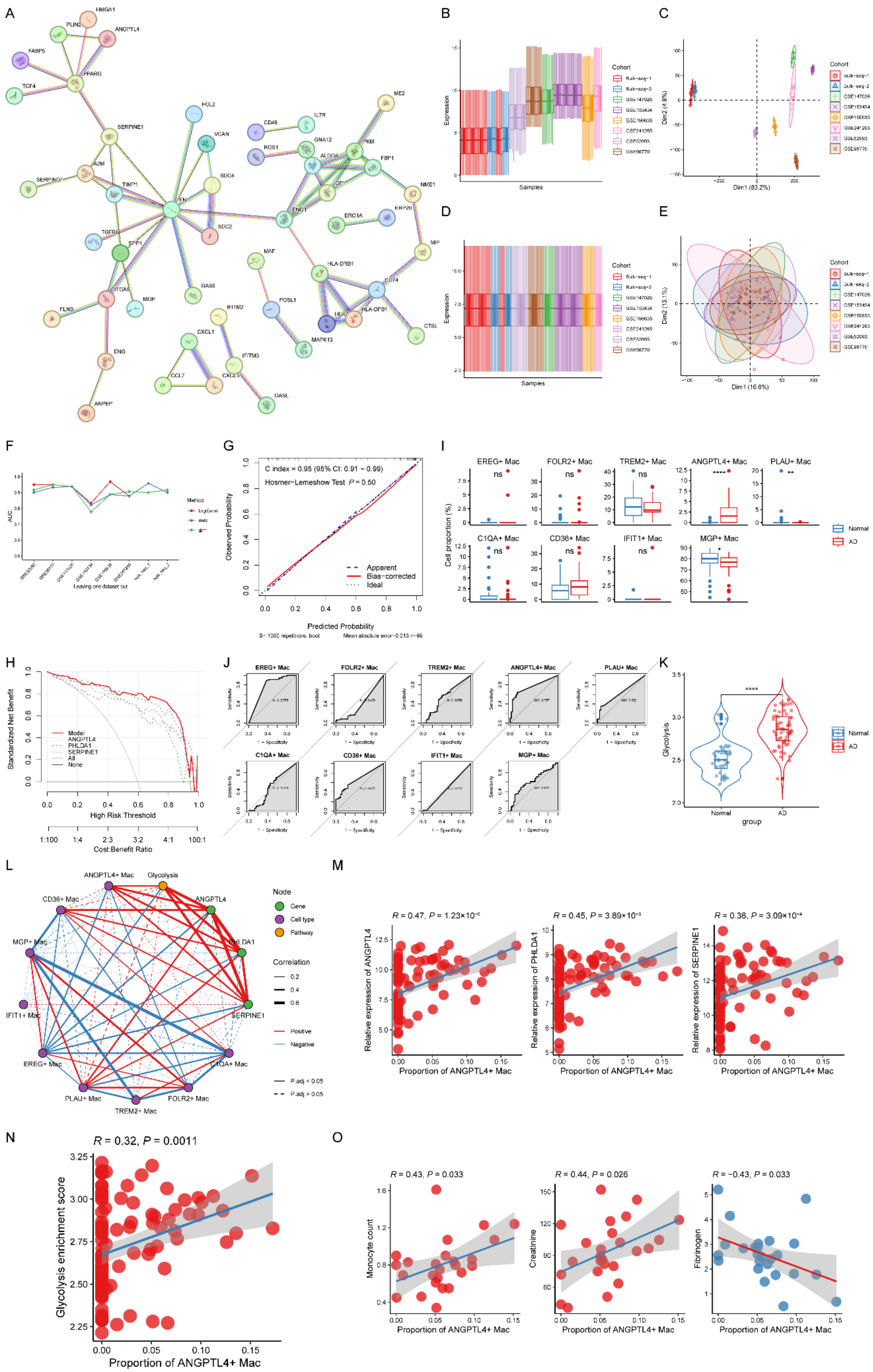

Supplementary Figure 6. Additional bulk RNA-seq analyses. (A) PPI network of the 119 intersected genes. (B) Boxplots of the eight datasets before adjusting batch effect. (C) PCA plot of the eight datasets before adjusting batch effect. (D) Boxplots of the eight datasets after adjusting batch effect. (E) PCA plot of the eight datasets after adjusting batch effect. (F) The AUC values of leave-one-cohort-out validation. (G) Calibration curve of the multivariate logistic regression model based on the three signatures. (H) Clinical decision curve of clinical prediction model. (I) Re-estimated macrophage proportions using leave-one-dataset-out reference. (J) ROC curves of the deconvolution-derived cell proportions of each macrophage subpopulation. (K) Difference in glycolysis scores between normal and AD samples (**** $P < 0.0001$). (L) Correlation network of the three signatures, deconvolution-derived macrophage proportions, and glycolysis scores. (M) Correlation between the deconvolution-derived cell proportion of ANGPTL4+ Mac and the three signatures. (N) Correlation between the deconvolution-derived cell proportion of ANGPTL4+ Mac and the glycolysis score. (O) Correlation between inferred ANGPTL4+ Mac proportion and clinical data of AD patients. Abbreviations: bulk RNA-seq, bulk RNA sequencing; PPI, protein-protein interaction; PCA, principal component analysis; AUC, area under curve; ROC, receiver operating characteristic; AD, aortic dissection.

1.2 Supplementary Tables

Supplementary Table 1. The primers used in this study.

Gene	Type	Sequence (5'→3')
ANGPTL4	Forward	GGCTCAGTGGACTTCAACCG
ANGPTL4	Reverse	CCGTGATGCTATGCACCTTCT
SERPINE1	Forward	GCACCACAGACGCGATCTT
SERPINE1	Reverse	ACCTCTGAAAAGTCCACTTGC
PHLDA1	Forward	GGAGATCGACTTTCGGTGCC
PHLDA1	Reverse	GGCCTGACGATTCTTGTACTG
ACTB	Forward	CACCATTGGCAATGAGCGGTTC
ACTB	Reverse	AGGTCTTTGCGGATGTCCACGT

Supplementary Table 2. The deconvolution-derived cell compositions of bulk RNA-seq data.

Mixture	C1QA+	FOLR2+	TREM2+	PLAU+	EREG+	IFIT1+	MGP+	CD36+	ANGPTL4+	P-value	Correlation	RMSE
GSM1259275	0.30	0.00	0.09	0.00	0.00	0.00	0.52	0.00	0.09	0.00	0.13	1.09
GSM1259276	0.29	0.00	0.00	0.00	0.00	0.00	0.19	0.52	0.00	0.00	0.20	1.01
GSM1259277	0.27	0.00	0.45	0.00	0.02	0.00	0.26	0.00	0.00	0.00	0.12	1.10
GSM1259278	0.20	0.00	0.42	0.00	0.02	0.00	0.36	0.00	0.00	0.00	0.11	1.09
GSM1259279	0.21	0.00	0.03	0.00	0.00	0.00	0.74	0.00	0.02	0.00	0.15	1.08
GSM1259280	0.18	0.00	0.36	0.00	0.05	0.00	0.38	0.03	0.00	0.00	0.11	1.09
GSM1259281	0.29	0.00	0.24	0.00	0.01	0.00	0.46	0.00	0.00	0.00	0.16	1.08
GSM1259282	0.29	0.00	0.34	0.00	0.00	0.00	0.26	0.08	0.03	0.00	0.11	1.09
GSM1259283	0.43	0.00	0.35	0.00	0.00	0.00	0.20	0.02	0.00	0.00	0.14	1.11
GSM1259284	0.24	0.00	0.11	0.00	0.00	0.00	0.24	0.39	0.03	0.00	0.10	1.06
GSM1259285	0.36	0.00	0.12	0.00	0.00	0.00	0.52	0.00	0.00	0.00	0.18	1.08
GSM1259286	0.36	0.00	0.13	0.00	0.00	0.01	0.50	0.00	0.00	0.00	0.19	1.08
GSM2611536	0.24	0.00	0.23	0.00	0.00	0.00	0.28	0.24	0.00	0.00	0.11	1.07
GSM2611537	0.30	0.00	0.00	0.05	0.00	0.00	0.36	0.30	0.00	0.00	0.12	1.07
GSM2611538	0.31	0.00	0.05	0.00	0.00	0.00	0.41	0.18	0.05	0.00	0.13	1.07
GSM2611539	0.27	0.00	0.20	0.00	0.00	0.00	0.53	0.00	0.00	0.00	0.14	1.08
GSM2611540	0.35	0.00	0.31	0.00	0.00	0.00	0.34	0.00	0.00	0.00	0.11	1.11
GSM2611541	0.26	0.00	0.17	0.00	0.00	0.00	0.30	0.27	0.00	0.00	0.12	1.07
GSM2611542	0.27	0.00	0.11	0.00	0.00	0.00	0.61	0.02	0.00	0.00	0.15	1.08
GSM2611543	0.22	0.00	0.17	0.00	0.00	0.00	0.59	0.02	0.00	0.00	0.16	1.07
GSM2611544	0.30	0.00	0.24	0.00	0.01	0.00	0.29	0.16	0.00	0.00	0.15	1.07
GSM2611545	0.24	0.00	0.25	0.00	0.01	0.00	0.31	0.18	0.00	0.00	0.12	1.07
GSM2611546	0.22	0.00	0.08	0.00	0.00	0.00	0.49	0.20	0.00	0.00	0.17	1.05
GSM4412476	0.00	0.16	0.00	0.00	0.00	0.00	0.84	0.00	0.00	0.00	0.10	1.10
GSM4412477	0.00	0.05	0.00	0.06	0.00	0.00	0.89	0.00	0.00	0.00	0.17	1.06
GSM4412478	0.00	0.31	0.00	0.02	0.01	0.00	0.66	0.00	0.00	0.00	0.08	1.12
GSM4412479	0.00	0.04	0.00	0.00	0.04	0.00	0.92	0.00	0.00	0.00	0.09	1.11
GSM4412480	0.00	0.31	0.00	0.06	0.00	0.00	0.63	0.00	0.00	0.00	0.11	1.11
GSM4412481	0.00	0.39	0.00	0.02	0.00	0.00	0.57	0.01	0.00	0.00	0.10	1.12

GSM4412482	0.00	0.00	0.00	0.08	0.00	0.00	0.91	0.01	0.00	0.00	0.11	1.09
GSM4412483	0.00	0.33	0.00	0.09	0.00	0.00	0.57	0.00	0.00	0.00	0.04	1.15
GSM4644214	0.00	0.00	0.00	0.06	0.00	0.00	0.90	0.00	0.04	0.00	0.19	1.05
GSM4644215	0.00	0.12	0.00	0.05	0.00	0.00	0.82	0.00	0.00	0.00	0.16	1.07
GSM4644216	0.00	0.18	0.00	0.05	0.00	0.00	0.74	0.02	0.02	0.00	0.15	1.07
GSM4644217	0.00	0.28	0.00	0.05	0.00	0.00	0.67	0.00	0.00	0.00	0.16	1.08
GSM4644218	0.00	0.04	0.00	0.03	0.00	0.00	0.86	0.00	0.07	0.00	0.17	1.05
GSM4644219	0.00	0.12	0.00	0.05	0.00	0.00	0.80	0.03	0.00	0.00	0.15	1.07
GSM4644220	0.00	0.09	0.00	0.07	0.00	0.00	0.67	0.00	0.17	0.00	0.16	1.07
GSM4644221	0.00	0.00	0.00	0.01	0.00	0.00	0.87	0.00	0.12	0.00	0.15	1.06
GSM4644222	0.00	0.14	0.00	0.04	0.00	0.00	0.81	0.01	0.00	0.00	0.16	1.06
GSM4644223	0.00	0.14	0.00	0.02	0.00	0.00	0.84	0.00	0.00	0.00	0.22	1.03
GSM4644224	0.00	0.04	0.00	0.00	0.05	0.00	0.91	0.00	0.00	0.00	0.17	1.07
GSM4644225	0.00	0.16	0.00	0.00	0.04	0.00	0.81	0.00	0.00	0.00	0.17	1.07
GSM4644226	0.00	0.17	0.00	0.00	0.03	0.00	0.80	0.00	0.00	0.00	0.16	1.07
GSM4644227	0.00	0.27	0.00	0.00	0.02	0.00	0.71	0.00	0.00	0.00	0.17	1.07
GSM4644228	0.00	0.24	0.00	0.00	0.06	0.00	0.70	0.00	0.00	0.00	0.14	1.09
GSM4644229	0.00	0.09	0.00	0.00	0.02	0.00	0.89	0.00	0.00	0.00	0.14	1.08
GSM4644230	0.00	0.09	0.00	0.00	0.09	0.00	0.80	0.02	0.00	0.00	0.14	1.09
GSM4644231	0.00	0.20	0.00	0.00	0.05	0.00	0.74	0.00	0.00	0.00	0.15	1.08
GSM4644232	0.00	0.16	0.00	0.00	0.02	0.00	0.82	0.00	0.00	0.00	0.18	1.06
GSM4644233	0.00	0.17	0.00	0.00	0.07	0.00	0.76	0.00	0.00	0.00	0.17	1.08
GSM5726864	0.03	0.00	0.22	0.00	0.03	0.00	0.71	0.00	0.00	0.00	0.08	1.10
GSM5726865	0.06	0.00	0.40	0.00	0.05	0.00	0.49	0.00	0.00	0.00	0.05	1.11
GSM5726866	0.00	0.00	0.36	0.00	0.02	0.00	0.62	0.00	0.00	0.00	0.07	1.09
GSM5726867	0.00	0.00	0.20	0.00	0.00	0.42	0.38	0.00	0.00	0.00	0.07	1.08
GSM5726868	0.00	0.00	0.27	0.00	0.05	0.02	0.66	0.00	0.00	0.00	0.07	1.10
GSM5726869	0.08	0.00	0.41	0.00	0.02	0.00	0.49	0.00	0.00	0.00	0.06	1.10
GSM5726870	0.00	0.00	0.53	0.00	0.00	0.00	0.30	0.15	0.02	0.00	0.07	1.07
GSM5726871	0.00	0.00	0.62	0.00	0.06	0.07	0.25	0.00	0.00	0.00	0.07	1.10
GSM7720285	0.00	0.00	0.00	0.00	0.00	0.00	0.86	0.00	0.14	0.00	0.23	1.02
GSM7720286	0.05	0.00	0.42	0.00	0.00	0.00	0.43	0.07	0.03	0.00	0.10	1.07
GSM7720287	0.00	0.00	0.22	0.00	0.00	0.00	0.75	0.00	0.03	0.00	0.20	1.03

GSM7720288	0.37	0.17	0.00	0.01	0.00	0.00	0.42	0.00	0.03	0.00	0.23	1.07
GSM7720289	0.38	0.12	0.00	0.00	0.03	0.00	0.48	0.00	0.00	0.00	0.19	1.09
GSM7720290	0.35	0.00	0.00	0.00	0.00	0.00	0.48	0.07	0.10	0.00	0.12	1.10
Control1_1	0.43	0.00	0.53	0.00	0.00	0.00	0.04	0.00	0.00	0.00	0.13	1.12
Control1_2	0.45	0.00	0.12	0.00	0.00	0.00	0.44	0.00	0.00	0.00	0.14	1.11
Control1_3	0.29	0.00	0.21	0.06	0.00	0.00	0.38	0.00	0.07	0.00	0.18	1.07
Control1_4	0.48	0.00	0.34	0.00	0.00	0.00	0.17	0.02	0.00	0.00	0.10	1.14
AD1_1	0.28	0.00	0.20	0.00	0.00	0.00	0.14	0.38	0.00	0.00	0.08	1.07
AD1_2	0.40	0.00	0.31	0.00	0.00	0.00	0.28	0.00	0.02	0.00	0.12	1.11
AD1_3	0.39	0.00	0.43	0.00	0.00	0.00	0.13	0.04	0.00	0.00	0.13	1.10
AD1_4	0.38	0.00	0.22	0.00	0.00	0.00	0.35	0.00	0.05	0.00	0.12	1.11
AD1_5	0.30	0.00	0.02	0.01	0.00	0.00	0.26	0.33	0.08	0.00	0.10	1.07
AD1_6	0.35	0.00	0.34	0.00	0.00	0.00	0.18	0.07	0.05	0.00	0.12	1.09
AD1_7	0.30	0.00	0.22	0.00	0.00	0.00	0.16	0.31	0.01	0.00	0.08	1.08
AD1_8	0.24	0.00	0.17	0.01	0.00	0.00	0.53	0.00	0.06	0.00	0.13	1.09
AD1_9	0.26	0.00	0.12	0.00	0.00	0.00	0.12	0.41	0.10	0.00	0.09	1.07
AD1_10	0.24	0.00	0.25	0.03	0.00	0.00	0.29	0.07	0.13	0.00	0.11	1.09
AD1_11	0.31	0.00	0.16	0.00	0.00	0.00	0.32	0.15	0.06	0.00	0.12	1.08
AD1_12	0.37	0.00	0.12	0.00	0.00	0.00	0.44	0.00	0.07	0.00	0.13	1.10
AD1_13	0.28	0.00	0.00	0.05	0.00	0.00	0.61	0.00	0.05	0.00	0.12	1.11
AD1_14	0.38	0.00	0.32	0.00	0.00	0.00	0.10	0.08	0.11	0.00	0.09	1.11
AD1_15	0.38	0.00	0.35	0.00	0.00	0.00	0.26	0.00	0.00	0.00	0.12	1.11
AD1_16	0.30	0.00	0.12	0.02	0.00	0.00	0.29	0.22	0.05	0.00	0.11	1.08
AD1_17	0.28	0.00	0.17	0.00	0.00	0.00	0.11	0.38	0.07	0.00	0.08	1.08
AD1_18	0.29	0.00	0.02	0.00	0.00	0.00	0.28	0.37	0.03	0.00	0.25	1.00
AD1_19	0.27	0.00	0.00	0.04	0.00	0.00	0.49	0.04	0.15	0.00	0.14	1.08
Control2_1	0.38	0.00	0.08	0.01	0.00	0.00	0.53	0.00	0.00	0.00	0.13	1.11
Control2_2	0.60	0.00	0.05	0.00	0.00	0.00	0.34	0.00	0.00	0.00	0.19	1.12
Control2_3	0.39	0.00	0.09	0.00	0.00	0.00	0.52	0.00	0.00	0.00	0.16	1.10
Control2_4	0.59	0.00	0.25	0.00	0.00	0.00	0.15	0.00	0.00	0.00	0.27	1.06
Control2_5	0.37	0.00	0.27	0.00	0.01	0.00	0.36	0.00	0.00	0.00	0.15	1.10
AD2_1	0.19	0.00	0.19	0.00	0.00	0.00	0.32	0.25	0.05	0.00	0.10	1.06
AD2_2	0.15	0.00	0.18	0.00	0.00	0.00	0.37	0.25	0.06	0.00	0.12	1.05

AD2_3	0.32	0.00	0.13	0.00	0.00	0.00	0.45	0.00	0.10	0.00	0.14	1.09
AD2_4	0.30	0.00	0.23	0.00	0.00	0.00	0.20	0.21	0.07	0.00	0.09	1.09
AD2_5	0.29	0.00	0.08	0.02	0.00	0.00	0.41	0.13	0.08	0.00	0.13	1.08
AD2_6	0.18	0.00	0.22	0.00	0.00	0.00	0.53	0.02	0.05	0.00	0.17	1.05

Supplementary Table 3. Detailed metrics of 102 machine learning algorithms.

Algorithm	AUCLower	AUC	AUCUpper	Accuracy	Kappa	AccuracyLower	AccuracyUpper	AccuracyNull	AccuracyPValue	McnemarPValue
ada	0.76	0.89	1.00	0.10	-0.72	0.02	0.27	0.59	1.00	0.33
AdaBag	0.79	0.90	1.00	0.90	0.79	0.73	0.98	0.59	0.00	1.00
adaboost	0.83	0.93	1.00	0.93	0.86	0.77	0.99	0.59	0.00	1.00
AdaBoost.M1	0.83	0.93	1.00	0.93	0.86	0.77	0.99	0.59	0.00	1.00
avNNet	0.65	0.80	0.95	0.83	0.63	0.64	0.94	0.59	0.01	0.37
bagEarth	0.83	0.93	1.00	0.07	-0.81	0.01	0.23	0.59	1.00	0.44
bagEarthGCV	0.76	0.89	1.00	0.10	-0.72	0.02	0.27	0.59	1.00	0.33
bagFDA	0.76	0.89	1.00	0.90	0.78	0.73	0.98	0.59	0.00	1.00
bagFDAGCV	0.76	0.89	1.00	0.90	0.78	0.73	0.98	0.59	0.00	1.00
bayesglm	0.65	0.80	0.95	0.83	0.63	0.64	0.94	0.59	0.01	0.37
blackboost	0.65	0.80	0.95	0.83	0.63	0.64	0.94	0.59	0.01	0.37
C5.0	0.75	0.88	1.00	0.90	0.78	0.73	0.98	0.59	0.00	0.25
C5.0Rules	0.68	0.83	0.97	0.83	0.65	0.64	0.94	0.59	0.01	1.00
C5.0Tree	0.68	0.83	0.97	0.83	0.65	0.64	0.94	0.59	0.01	1.00
cforest	0.71	0.85	0.99	0.86	0.71	0.68	0.96	0.59	0.00	0.62
ctree	0.65	0.80	0.95	0.79	0.58	0.60	0.92	0.59	0.02	0.68
ctree2	0.65	0.80	0.95	0.79	0.58	0.60	0.92	0.59	0.02	0.68
dnn	0.50	0.50	0.50	0.59	0.00	0.39	0.76	0.59	0.58	0.00
dwdLinear	0.83	0.93	1.00	0.93	0.86	0.77	0.99	0.59	0.00	1.00
dwdPoly	0.50	0.50	0.50	0.59	0.00	0.39	0.76	0.59	0.58	0.00
dwdRadial	0.71	0.85	0.99	0.86	0.71	0.68	0.96	0.59	0.00	0.62
earth	0.81	0.92	1.00	0.93	0.85	0.77	0.99	0.59	0.00	0.48
evtree	0.78	0.88	0.99	0.86	0.73	0.68	0.96	0.59	0.00	0.13
extraTrees	0.76	0.89	1.00	0.90	0.78	0.73	0.98	0.59	0.00	1.00
fda	0.81	0.92	1.00	0.93	0.85	0.77	0.99	0.59	0.00	0.48
gamboost	0.76	0.89	1.00	0.90	0.78	0.73	0.98	0.59	0.00	1.00

gamSpline	0.35	0.54	0.72	0.52	0.07	0.33	0.71	0.59	0.83	0.18
gaussprLinear	0.72	0.86	0.99	0.86	0.72	0.68	0.96	0.59	0.00	1.00
gaussprRadial	0.71	0.85	0.99	0.86	0.71	0.68	0.96	0.59	0.00	0.62
gbm	0.88	0.96	1.00	0.97	0.93	0.82	1.00	0.59	0.00	1.00
gcvEarth	0.52	0.68	0.84	0.72	0.39	0.53	0.87	0.59	0.09	0.08
glm	0.40	0.59	0.77	0.59	0.17	0.39	0.76	0.59	0.58	0.77
glmboost	0.83	0.93	1.00	0.93	0.86	0.77	0.99	0.59	0.00	1.00
glmnet	0.76	0.89	1.00	0.90	0.78	0.73	0.98	0.59	0.00	1.00
glmStepAIC	0.45	0.61	0.77	0.66	0.23	0.46	0.82	0.59	0.29	0.11
gpls	0.35	0.54	0.73	0.55	0.09	0.36	0.74	0.59	0.72	1.00
hda	0.76	0.89	1.00	0.90	0.78	0.73	0.98	0.59	0.00	1.00
hdda	0.68	0.83	0.97	0.83	0.65	0.64	0.94	0.59	0.01	1.00
J48	0.65	0.80	0.95	0.79	0.58	0.60	0.92	0.59	0.02	0.68
JRip	0.75	0.88	1.00	0.90	0.78	0.73	0.98	0.59	0.00	0.25
kknn	0.83	0.93	1.00	0.93	0.86	0.77	0.99	0.59	0.00	1.00
knn	0.72	0.86	0.99	0.86	0.72	0.68	0.96	0.59	0.00	1.00
lda	0.52	0.69	0.86	0.72	0.40	0.53	0.87	0.59	0.09	0.29
lda2	0.52	0.69	0.86	0.72	0.40	0.53	0.87	0.59	0.09	0.29
LMT	0.71	0.85	0.99	0.86	0.71	0.68	0.96	0.59	0.00	0.62
LogitBoost	1.00	1.00	1.00	1.00	1.00	0.88	1.00	0.59	0.00	
mlp	0.76	0.89	1.00	0.90	0.78	0.73	0.98	0.59	0.00	1.00
mlpML	0.71	0.85	0.99	0.86	0.71	0.68	0.96	0.59	0.00	0.62
mlpSGD	0.83	0.93	1.00	0.93	0.86	0.77	0.99	0.59	0.00	1.00
mlpWeightDecayML	0.50	0.50	0.50	0.59	0.00	0.39	0.76	0.59	0.58	0.00
monmlp	0.67	0.82	0.97	0.83	0.64	0.64	0.94	0.59	0.01	1.00
multinom	0.75	0.87	0.99	0.86	0.72	0.68	0.96	0.59	0.00	0.62
naive_bayes	0.86	0.94	1.00	0.93	0.86	0.77	0.99	0.59	0.00	0.48
nb	0.86	0.94	1.00	0.93	0.86	0.77	0.99	0.59	0.00	0.48
nnet	0.76	0.89	1.00	0.90	0.78	0.73	0.98	0.59	0.00	1.00
nodeHarvest	0.83	0.93	1.00	0.93	0.86	0.77	0.99	0.59	0.00	1.00
null	0.50	0.50	0.50	0.59	0.00	0.39	0.76	0.59	0.58	0.00
OneR	0.75	0.88	1.00	0.90	0.78	0.73	0.98	0.59	0.00	0.25
pam	0.82	0.91	1.00	0.90	0.79	0.73	0.98	0.59	0.00	0.25

parRF	0.76	0.89	1.00	0.90	0.78	0.73	0.98	0.59	0.00	1.00
PART	0.76	0.89	1.00	0.90	0.78	0.73	0.98	0.59	0.00	1.00
pcaNNet	0.65	0.80	0.95	0.83	0.63	0.64	0.94	0.59	0.01	0.37
pda	0.32	0.51	0.71	0.52	0.03	0.33	0.71	0.59	0.83	0.79
pda2	0.76	0.89	1.00	0.90	0.78	0.73	0.98	0.59	0.00	1.00
plr	0.76	0.89	1.00	0.90	0.78	0.73	0.98	0.59	0.00	1.00
plsRglm	0.76	0.89	1.00	0.90	0.78	0.73	0.98	0.59	0.00	1.00
PRIM	0.63	0.79	0.95	0.79	0.57	0.60	0.92	0.59	0.02	1.00
ranger	0.76	0.89	1.00	0.90	0.78	0.73	0.98	0.59	0.00	1.00
rbfDDA	0.46	0.54	0.62	0.62	0.10	0.42	0.79	0.59	0.43	0.00
Rborist	0.83	0.93	1.00	0.93	0.86	0.77	0.99	0.59	0.00	1.00
rda	0.79	0.90	1.00	0.90	0.79	0.73	0.98	0.59	0.00	1.00
regLogistic	0.78	0.88	0.99	0.86	0.73	0.68	0.96	0.59	0.00	0.13
rf	0.76	0.89	1.00	0.90	0.78	0.73	0.98	0.59	0.00	1.00
rmda	0.91	0.97	1.00	0.97	0.93	0.82	1.00	0.59	0.00	1.00
rotationForest	0.76	0.89	1.00	0.90	0.78	0.73	0.98	0.59	0.00	1.00
rotationForestCp	0.83	0.93	1.00	0.93	0.86	0.77	0.99	0.59	0.00	1.00
rpart	0.75	0.88	1.00	0.90	0.78	0.73	0.98	0.59	0.00	0.25
rpart1SE	0.75	0.88	1.00	0.90	0.78	0.73	0.98	0.59	0.00	0.25
rpart2	0.75	0.88	1.00	0.90	0.78	0.73	0.98	0.59	0.00	0.25
RRF	0.83	0.93	1.00	0.93	0.86	0.77	0.99	0.59	0.00	1.00
RRFglobal	0.76	0.89	1.00	0.90	0.78	0.73	0.98	0.59	0.00	1.00
rrlda	0.47	0.66	0.84	0.66	0.31	0.46	0.82	0.59	0.29	0.75
sda	0.83	0.93	1.00	0.93	0.86	0.77	0.99	0.59	0.00	1.00
sdwd	0.50	0.50	0.50	0.59	0.00	0.39	0.76	0.59	0.58	0.00
slda	0.83	0.93	1.00	0.93	0.86	0.77	0.99	0.59	0.00	1.00
sparseLDA	0.83	0.93	1.00	0.93	0.86	0.77	0.99	0.59	0.00	1.00
spls	0.61	0.77	0.93	0.79	0.56	0.60	0.92	0.59	0.02	0.68
stepLDA	0.72	0.86	0.99	0.86	0.72	0.68	0.96	0.59	0.00	1.00
stepQDA	0.78	0.88	0.99	0.86	0.73	0.68	0.96	0.59	0.00	0.13
svmLinear	0.65	0.80	0.95	0.83	0.63	0.64	0.94	0.59	0.01	0.37
svmLinear2	0.65	0.80	0.95	0.83	0.63	0.64	0.94	0.59	0.01	0.37
svmLinearWeights	0.65	0.80	0.95	0.83	0.63	0.64	0.94	0.59	0.01	0.37

svmPoly	0.78	0.88	0.99	0.86	0.73	0.68	0.96	0.59	0.00	0.13
svmRadial	0.76	0.89	1.00	0.90	0.78	0.73	0.98	0.59	0.00	1.00
svmRadialCost	0.71	0.85	0.99	0.86	0.71	0.68	0.96	0.59	0.00	0.62
svmRadialSigma	0.76	0.89	1.00	0.90	0.78	0.73	0.98	0.59	0.00	1.00
svmRadialWeights	0.65	0.80	0.95	0.83	0.63	0.64	0.94	0.59	0.01	0.37
treebag	0.76	0.89	1.00	0.90	0.78	0.73	0.98	0.59	0.00	1.00
wsrf	0.76	0.89	1.00	0.90	0.78	0.73	0.98	0.59	0.00	1.00
xgbDART	0.83	0.93	1.00	0.93	0.86	0.77	0.99	0.59	0.00	1.00
xgbLinear	0.81	0.92	1.00	0.93	0.85	0.77	0.99	0.59	0.00	0.48
xgbTree	0.79	0.90	1.00	0.90	0.79	0.73	0.98	0.59	0.00	1.00

Supplementary Table 4. Correlation between deconvoluted macrophage proportions and clinical parameters.

var1	var2	cor	cor_lci	cor_uci	pval	padj
C1QA+ Mac	HR	0.02	-0.38	0.41	0.92	0.94
C1QA+ Mac	SBP	0.34	-0.06	0.65	0.10	0.39
C1QA+ Mac	DBP	0.04	-0.36	0.43	0.85	0.93
C1QA+ Mac	PP	0.42	0.03	0.70	0.04	0.23
C1QA+ Mac	MAP	0.19	-0.22	0.55	0.36	0.60
C1QA+ Mac	W	0.43	0.04	0.71	0.03	0.23
C1QA+ Mac	H	0.34	-0.07	0.65	0.10	0.39
C1QA+ Mac	BMI	0.26	-0.15	0.59	0.22	0.60
C1QA+ Mac	hospitalStay	0.05	-0.36	0.43	0.83	0.93
C1QA+ Mac	icuStay	-0.02	-0.42	0.37	0.91	0.94
C1QA+ Mac	postOperationStay	-0.19	-0.54	0.22	0.36	0.60
C1QA+ Mac	operationDuration	-0.20	-0.55	0.21	0.34	0.60
C1QA+ Mac	WBC	-0.38	-0.67	0.02	0.06	0.32
C1QA+ Mac	PLT	-0.19	-0.54	0.23	0.38	0.60
C1QA+ Mac	NEUT	-0.37	-0.67	0.03	0.07	0.33
C1QA+ Mac	LYM	-0.26	-0.59	0.15	0.21	0.60
C1QA+ Mac	MONO	-0.08	-0.46	0.33	0.71	0.87
C1QA+ Mac	Alb	-0.40	-0.68	0.00	0.05	0.30
C1QA+ Mac	ALT	0.04	-0.36	0.43	0.84	0.93
C1QA+ Mac	AST	-0.03	-0.42	0.37	0.87	0.94
C1QA+ Mac	Crea	-0.17	-0.53	0.25	0.43	0.66
C1QA+ Mac	PT	-0.19	-0.54	0.22	0.37	0.60
C1QA+ Mac	APTT	-0.01	-0.40	0.39	0.96	0.96
C1QA+ Mac	Fbg	0.39	-0.01	0.68	0.06	0.32
TREM2+ Mac	HR	-0.04	-0.43	0.36	0.84	0.93
TREM2+ Mac	SBP	0.06	-0.35	0.44	0.78	0.93
TREM2+ Mac	DBP	0.00	-0.39	0.40	0.99	0.99
TREM2+ Mac	PP	0.08	-0.33	0.46	0.72	0.87
TREM2+ Mac	MAP	0.03	-0.37	0.42	0.89	0.94
TREM2+ Mac	W	0.21	-0.20	0.56	0.31	0.60
TREM2+ Mac	H	0.20	-0.21	0.55	0.34	0.60
TREM2+ Mac	BMI	0.09	-0.32	0.47	0.67	0.84
TREM2+ Mac	hospitalStay	0.43	0.04	0.71	0.03	0.23
TREM2+ Mac	icuStay	0.13	-0.28	0.50	0.54	0.75
TREM2+ Mac	postOperationStay	0.24	-0.17	0.58	0.25	0.60
TREM2+ Mac	operationDuration	-0.27	-0.60	0.14	0.19	0.55
TREM2+ Mac	WBC	-0.35	-0.66	0.05	0.09	0.36
TREM2+ Mac	PLT	0.20	-0.21	0.55	0.34	0.60
TREM2+ Mac	NEUT	-0.38	-0.67	0.02	0.06	0.32
TREM2+ Mac	LYM	-0.02	-0.41	0.38	0.92	0.94
TREM2+ Mac	MONO	-0.16	-0.52	0.25	0.44	0.67

TREM2+ Mac	Alb	0.06	-0.35	0.44	0.79	0.93
TREM2+ Mac	ALT	0.20	-0.21	0.55	0.33	0.60
TREM2+ Mac	AST	-0.21	-0.56	0.20	0.31	0.60
TREM2+ Mac	Crea	-0.53	-0.76	-0.17	0.01	0.10
TREM2+ Mac	PT	-0.54	-0.77	-0.18	0.01	0.10
TREM2+ Mac	APTT	-0.11	-0.48	0.30	0.60	0.79
TREM2+ Mac	Fbg	0.59	0.25	0.80	0.00	0.07
PLAU+ Mac	HR	0.38	-0.02	0.67	0.06	0.32
PLAU+ Mac	SBP	-0.21	-0.56	0.20	0.32	0.60
PLAU+ Mac	DBP	-0.14	-0.50	0.27	0.52	0.74
PLAU+ Mac	PP	-0.17	-0.53	0.24	0.43	0.66
PLAU+ Mac	MAP	-0.18	-0.54	0.23	0.38	0.60
PLAU+ Mac	W	-0.14	-0.51	0.27	0.51	0.74
PLAU+ Mac	H	0.22	-0.20	0.56	0.30	0.60
PLAU+ Mac	BMI	-0.29	-0.61	0.12	0.16	0.50
PLAU+ Mac	hospitalStay	-0.18	-0.54	0.23	0.38	0.60
PLAU+ Mac	icuStay	-0.14	-0.51	0.27	0.49	0.73
PLAU+ Mac	postOperationStay	-0.12	-0.49	0.29	0.58	0.78
PLAU+ Mac	operationDuration	0.20	-0.21	0.55	0.34	0.60
PLAU+ Mac	WBC	0.46	0.08	0.72	0.02	0.23
PLAU+ Mac	PLT	-0.49	-0.74	-0.12	0.01	0.18
PLAU+ Mac	NEUT	0.48	0.10	0.73	0.02	0.19
PLAU+ Mac	LYM	-0.03	-0.42	0.37	0.90	0.94
PLAU+ Mac	MONO	0.61	0.29	0.81	0.00	0.07
PLAU+ Mac	Alb	-0.27	-0.60	0.14	0.19	0.55
PLAU+ Mac	ALT	-0.22	-0.57	0.19	0.29	0.60
PLAU+ Mac	AST	0.32	-0.09	0.63	0.12	0.42
PLAU+ Mac	Crea	0.55	0.20	0.78	0.00	0.10
PLAU+ Mac	PT	0.54	0.19	0.77	0.01	0.10
PLAU+ Mac	APTT	-0.13	-0.50	0.28	0.53	0.75
PLAU+ Mac	Fbg	-0.41	-0.69	-0.02	0.04	0.25
MGP+ Mac	HR	0.33	-0.07	0.64	0.10	0.39
MGP+ Mac	SBP	-0.20	-0.55	0.21	0.34	0.60
MGP+ Mac	DBP	-0.18	-0.54	0.23	0.38	0.60
MGP+ Mac	PP	-0.12	-0.49	0.29	0.58	0.78
MGP+ Mac	MAP	-0.21	-0.56	0.20	0.32	0.60
MGP+ Mac	W	-0.57	-0.79	-0.22	0.00	0.09
MGP+ Mac	H	-0.20	-0.55	0.21	0.34	0.60
MGP+ Mac	BMI	-0.49	-0.74	-0.11	0.01	0.18
MGP+ Mac	hospitalStay	-0.20	-0.55	0.21	0.34	0.60
MGP+ Mac	icuStay	-0.04	-0.43	0.36	0.84	0.93
MGP+ Mac	postOperationStay	-0.05	-0.44	0.35	0.81	0.93
MGP+ Mac	operationDuration	-0.11	-0.48	0.30	0.60	0.79
MGP+ Mac	WBC	0.59	0.25	0.80	0.00	0.07
MGP+ Mac	PLT	-0.19	-0.54	0.22	0.36	0.60
MGP+ Mac	NEUT	0.59	0.26	0.80	0.00	0.07

MGP+ Mac	LYM	0.29	-0.12	0.62	0.16	0.50
MGP+ Mac	MONO	0.21	-0.21	0.56	0.32	0.60
MGP+ Mac	Alb	0.13	-0.28	0.50	0.53	0.75
MGP+ Mac	ALT	0.03	-0.37	0.42	0.88	0.94
MGP+ Mac	AST	0.45	0.06	0.72	0.02	0.23
MGP+ Mac	Crea	0.44	0.05	0.71	0.03	0.23
MGP+ Mac	PT	0.43	0.04	0.70	0.03	0.23
MGP+ Mac	APTT	-0.28	-0.61	0.13	0.18	0.54
MGP+ Mac	Fbg	-0.22	-0.57	0.19	0.28	0.60
CD36+ Mac	HR	-0.32	-0.63	0.09	0.12	0.42
CD36+ Mac	SBP	0.08	-0.33	0.46	0.71	0.87
CD36+ Mac	DBP	0.15	-0.26	0.52	0.47	0.70
CD36+ Mac	PP	-0.02	-0.41	0.38	0.92	0.94
CD36+ Mac	MAP	0.13	-0.28	0.50	0.54	0.75
CD36+ Mac	W	0.31	-0.10	0.63	0.13	0.44
CD36+ Mac	H	-0.07	-0.45	0.34	0.75	0.90
CD36+ Mac	BMI	0.38	-0.02	0.67	0.06	0.32
CD36+ Mac	hospitalStay	-0.16	-0.52	0.25	0.45	0.68
CD36+ Mac	icuStay	-0.06	-0.44	0.35	0.79	0.93
CD36+ Mac	postOperationStay	-0.04	-0.43	0.36	0.84	0.93
CD36+ Mac	operationDuration	0.33	-0.08	0.64	0.11	0.41
CD36+ Mac	WBC	-0.26	-0.59	0.15	0.22	0.60
CD36+ Mac	PLT	0.25	-0.16	0.59	0.23	0.60
CD36+ Mac	NEUT	-0.24	-0.58	0.17	0.24	0.60
CD36+ Mac	LYM	-0.16	-0.52	0.25	0.45	0.68
CD36+ Mac	MONO	-0.22	-0.57	0.19	0.29	0.60
CD36+ Mac	Alb	0.09	-0.32	0.47	0.67	0.84
CD36+ Mac	ALT	-0.20	-0.55	0.21	0.34	0.60
CD36+ Mac	AST	-0.36	-0.66	0.04	0.08	0.34
CD36+ Mac	Crea	-0.13	-0.50	0.28	0.54	0.75
CD36+ Mac	PT	-0.08	-0.46	0.33	0.72	0.87
CD36+ Mac	APTT	0.32	-0.09	0.63	0.12	0.42
CD36+ Mac	Fbg	-0.25	-0.59	0.16	0.22	0.60
ANGPTL4+ Mac	HR	-0.11	-0.48	0.30	0.60	0.79
ANGPTL4+ Mac	SBP	-0.22	-0.56	0.20	0.30	0.60
ANGPTL4+ Mac	DBP	0.10	-0.31	0.47	0.64	0.82
ANGPTL4+ Mac	PP	-0.37	-0.67	0.03	0.07	0.33
ANGPTL4+ Mac	MAP	-0.05	-0.44	0.35	0.82	0.93
ANGPTL4+ Mac	W	-0.33	-0.64	0.07	0.10	0.39
ANGPTL4+ Mac	H	-0.24	-0.58	0.17	0.25	0.60
ANGPTL4+ Mac	BMI	-0.19	-0.55	0.22	0.36	0.60
ANGPTL4+ Mac	hospitalStay	0.08	-0.32	0.46	0.69	0.87
ANGPTL4+ Mac	icuStay	0.10	-0.31	0.48	0.64	0.82
ANGPTL4+ Mac	postOperationStay	0.03	-0.37	0.42	0.90	0.94
ANGPTL4+ Mac	operationDuration	0.23	-0.18	0.58	0.26	0.60
ANGPTL4+ Mac	WBC	0.24	-0.17	0.58	0.25	0.60

ANGPTL4+ Mac	PLT	-0.31	-0.63	0.10	0.13	0.44
ANGPTL4+ Mac	NEUT	0.24	-0.17	0.58	0.25	0.60
ANGPTL4+ Mac	LYM	0.02	-0.38	0.41	0.93	0.95
ANGPTL4+ Mac	MONO	0.43	0.04	0.70	0.03	0.23
ANGPTL4+ Mac	Alb	-0.21	-0.56	0.20	0.31	0.60
ANGPTL4+ Mac	ALT	0.05	-0.35	0.44	0.82	0.93
ANGPTL4+ Mac	AST	0.23	-0.18	0.58	0.26	0.60
ANGPTL4+ Mac	Crea	0.44	0.06	0.71	0.03	0.23
ANGPTL4+ Mac	PT	0.36	-0.04	0.66	0.08	0.34
ANGPTL4+ Mac	APTT	0.24	-0.17	0.58	0.25	0.60
ANGPTL4+ Mac	Fbg	-0.43	-0.70	-0.04	0.03	0.23
